# Decolorization and Detoxification of Azo and Triphenylmethane Dyes Damaging Human Health by Crude Laccase from White-Rot Fungus *Pleurotus ostreatus* Yang1 and Molecular Docking Between Laccase and Structurally Diverse Dyes

**DOI:** 10.3390/ijms26178363

**Published:** 2025-08-28

**Authors:** Qingchen Li, Yuguo Feng, Siying Zhuang, Linman Kang, Yang Yang

**Affiliations:** Hubei Key Laboratory of Genetic Regulation and Integrative Biology, School of Life Sciences, Central China Normal University, Wuhan 430079, China; liqingchen227@163.com (Q.L.); fyg0029@163.com (Y.F.); 18060082686@163.com (S.Z.); kanglm0202@163.com (L.K.)

**Keywords:** *Pleurotus ostreatus*, laccase, azo dye, triphenylmethane dye, decolorization, detoxification, molecular docking

## Abstract

This study systematically investigated the decolorization efficacy and detoxification effect of crude laccase derived from *Pleurotus ostreatus* yang1 on azo and triphenylmethane dyes. This research encompassed decolorization efficiencies for 15 dyes (7 azo dyes and 8 triphenylmethane dyes), time course decolorization kinetics, and detoxification assessment using rice (*Oryza sativa*) and wheat (*Triticum aestivum*) seed germination as phytotoxicity indicators for both single-dye and mixed-dye systems. Molecular docking was employed to elucidate the laccase–dye interaction mechanisms. The results demonstrated that crude laccase from *Pleurotus ostreatus* yang1 exhibited significant decolorization efficiency and effective detoxification capacity toward both azo dyes and triphenylmethane dyes. It also displayed considerable decolorization efficiency for mixtures of azo and triphenylmethane dyes (mixture of two types of dyes), along with strong detoxification capability against the phytotoxicity of mixed dyes. Crude laccase showed robust continuous batch decolorization capability for azo dyes Alpha-naphthol Orange (α-NO) and Mordant Blue 13 (MB13). Similarly, it achieved high continuous batch decolorization efficiency for triphenylmethane dyes (e.g., Cresol Red, Acid Green 50) while maintaining stable laccase activity throughout the decolorization process. Crude laccase demonstrated excellent reusability and sustainable degradation performance during the continuous batch decolorization. The decolorization of crude laccase could significantly reduce or completely eliminate the phytotoxicity of both single dyes and mixtures of two dyes (pairwise mixtures of different types of dyes, totaling 18 different combinations). The results of molecular docking between the laccase protein and structurally diverse dyes further elucidated the underlying causes and potential mechanisms for variations in the catalytic ability of laccase toward different structural dyes. In summary, crude laccase from *Pleurotus ostreatus* yang1 possessed great application value and potential for efficiently degrading and detoxifying dye pollutants of different structural types.

## 1. Introduction

Laccase (EC 1.10.3.2) is a multicopper oxidase that catalyzes single-electron transfer reactions by oxidizing substrates such as phenolic compounds and amines, producing water as the sole byproduct, and is characterized by a high efficiency and environmental friendliness [1,2]. Laccase possesses a broad substrate specificity and can catalyze the oxidation of various substrates, including phenolic compounds, aromatic amines, alcohols, and others. The products of laccase-catalyzed reactions are non-polluting to the environment. Compared with chemical catalysts, the catalytic process of laccase is generally milder, and water is the only byproduct. Since the entire reaction process catalyzed by laccase generates only water as a byproduct and has a wide range of substrates, it serves as an environmentally friendly and green enzyme, possessing immense application value and potential in fields such as the bioremediation of refractory environmental pollutants [3,4,5].

Dyes are widely used in industries including textiles, dyeing, food, papermaking, printing, cosmetics, paints, plastics, and leather. Synthetic dyes can be classified into five categories based on their chromophores: azo dyes, anthraquinone dyes, indigoid dyes, triphenylmethane dyes, and phthalocyanine dyes. Among these, azo and triphenylmethane dyes are the two most commonly used types of industrial dyes [6]. Despite the immense application value of dyes with different chemical structures in modern industry, their widespread use has also led to serious environmental pollution problems [7,8]. Due to the extensive application of dyes across various industries, large volumes of dye-containing wastewater are discharged into the environment. These dye pollutants, characterized by a high toxicity, carcinogenicity, teratogenicity, and persistence, pose severe hazards to the ecological environment and human health [9,10]. The discharge of dye wastewater into water bodies reduces light transmittance and dissolved oxygen content and introduces toxic organic compounds. Nutrients such as nitrogen and phosphorus commonly present in dye wastewater have the potential to trigger eutrophication, which in turn can lead to the excessive proliferation of aquatic organisms such as algae. This not only intensifies the depletion of dissolved oxygen in the water body, but also severely impacts the growth and reproduction of various other aquatic organisms due to the disruption of the ecological balance [11,12,13]. Dyes can also cause serious damage to human health. They exert adverse effects on the human reproductive system, liver and kidney systems, and central nervous system, leading to a series of related diseases [14,15,16]. Therefore, the effective treatment of dye pollutants has become a crucial environmental issue requiring urgent resolution. Research on the efficient degradation of dye pollutants is of significant theoretical and practical value for the better protection of ecological environments such as water and soil, and for effectively eliminating the serious threats and harm posed by dye pollutants to human health.

Recent studies have indicated that laccase possesses considerable application value and potential in the decolorization of various types of industrial dyes [17,18,19,20]. The capability of laccase to catalyze dye decolorization was closely related to the structure of the dye. The decolorization efficiency of the same laccase varied for dyes with different structures was investigated [21]. Laccases from different sources also exhibited distinct differences in their abilities to catalyze the decolorization of the same dye. Factors such as laccase mediators, substrate concentration, enzyme activity, reaction temperature, pH, and ionic strength were important parameters influencing the dye degradation capability of laccase. The same laccase exhibited different substrate affinities for dyes with different structures [22,23]. Beyond decolorizing dyes, laccase can also catalyze the transformation of dye molecules into simpler and less toxic compounds [24,25]. Compared with using purified laccase, using crude laccase for dye decolorization can significantly reduce the cost in the practical treatment of dye pollutants. Crude laccase refers to the culture supernatant containing laccase activity, which is preliminarily isolated from fungal cultures with high laccase production. Its main component is laccase.

Previous research primarily concentrated on the decolorization and degradation of dyes using purified laccase. However, from a practical application perspective, obtaining purified laccase involved multiple steps, making the cost of using it for dye pollutant treatment relatively high. On the other hand, using crude laccase for dye degradation offered unique advantages such as a lower cost, simpler operating conditions, and easier accessibility, which can significantly reduce the cost of laccase in the practical treatment of dye pollutants. Therefore, to better explore and utilize the potential and application value of crude laccase in the degradation and detoxification of dye pollutants with different structural types, this study employed crude laccase from white-rot fungus *Pleurotus ostreatus* yang1 (hereinafter referred to as the yang1 strain) as the research material. Selecting the most industrially prevalent azo and triphenylmethane dyes, we systematically investigated the decolorization efficiency of crude laccase from the yang1 strain towards these two types of dyes, their time course decolorization curves, and degradation kinetics. We also studied the repeated-batch decolorization performance of laccase on azo and triphenylmethane dyes and further evaluated its detoxification capacity regarding the phytotoxicity of azo dyes, triphenylmethane dyes, and their mixtures (single dyes, mixed dyes). Additionally, molecular docking studies between the laccase protein and dye molecules of different structures were conducted to reveal the interaction mechanism between the laccase protein and dye molecules, aiming to theoretically explain the reasons and possible mechanisms underlying the differences in the catalytic capability of laccase towards dyes with different structures. Our research results play a positive role in promoting the practical application of fungal laccase in environmental protection and bioremediation, particularly in the efficient treatment of refractory environmental organic pollutants such as dye pollutants.

## 2. Results

### 2.1. Study on the Decolorization of Different Azo Dyes by Crude Laccase from the Yang1 Strain

#### 2.1.1. Measurement of the 24 H Decolorization Efficiencies of Different Azo Dyes Using Crude Laccase (1 U/mL, 2 U/mL)

A list of the full names and abbreviations of all dyes used in this study can be seen in Table 1.

**Figure 1:**
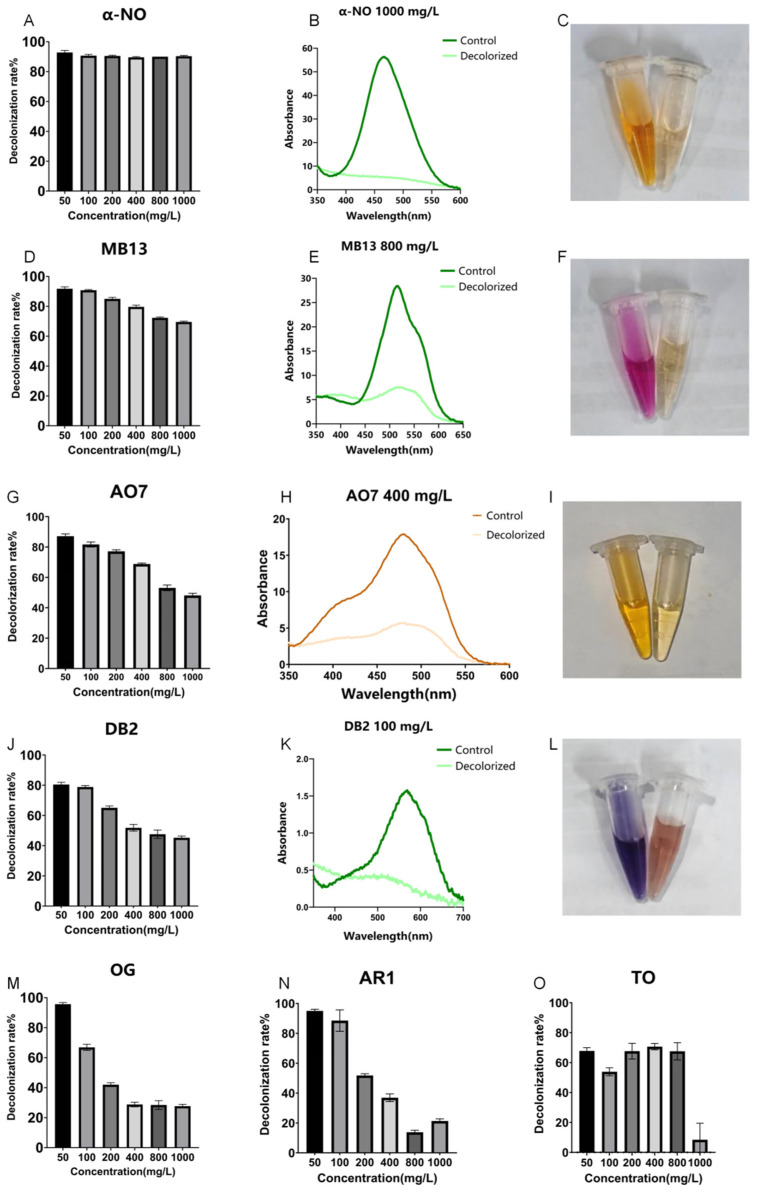
Decolorization of different azo dyes by crude laccase from the yang1 strain (1 U/mL) at 30 °C. (**A**). Decolorization efficiencies of different concentrations of azo dye α-NO treated with 1 U/mL crude laccase (24 h). (**B**). Spectral scanning graph of azo dye α-NO before and after treatment with 1 U/mL crude laccase (dye concentration: 1000 mg/L). (**C**). Color change diagram of azo dye α-NO before and after laccase decolorization (left—before decolorization, right—after decolorization). (**D**). Decolorization efficiencies of different concentrations of azo dye MB13 treated with 1 U/mL crude laccase (24 h). (**E**). Spectral scanning graph of azo dye MB13 before and after treatment with 1 U/mL crude laccase (dye concentration: 800 mg/L). (**F**). Color change diagram of azo dye MB13 before and after laccase decolorization (left—before decolorization, right—after decolorization). (**G**). Decolorization efficiencies of different concentrations of azo dye AO7 treated with 1 U/mL crude laccase (24 h). (**H**). Spectral scanning graph of azo dye AO7 before and after treatment with 1 U/mL crude laccase (dye concentration: 400 mg/L). (**I**). Color change diagram of azo dye AO7 before and after laccase decolorization (left—before decolorization, right—after decolorization). (**J**). Decolorization efficiencies of different concentrations of azo dye DB2 treated with 1 U/mL crude laccase (24 h). (**K**). Spectral scanning graph of azo dye DB2 before and after treatment with 1 U/mL crude laccase (dye concentration: 100 mg/L). (**L**). Color change diagram of azo dye DB2 before and after laccase decolorization (left—before decolorization, right—after decolorization). (**M**). Decolorization efficiencies of different concentrations of azo dye OG treated with 1 U/mL crude laccase (24 h). (**N**). Decolorization efficiencies of different concentrations of azo dye AR1 treated with 1 U/mL crude laccase (24 h). (**O**). Decolorization efficiencies of different concentrations of azo dye TO treated with 1 U/mL crude laccase (24 h).

As illustrated in Figure 1, crude laccase from the yang1 strain had an excellent decolorization effect on azo dyes α-NO, OG, and MB13, a good decolorization effect on DB2 and AO7, and some decolorization effect on AR1 and TO. The decolorization efficiencies of AR1 and TO decreased significantly as the concentration increased. Concentrations of 50, 100, 200, 400, 800, and 1000 mg/L of α-NO were successfully decolorized by crude laccase (1 U/mL). The 24 h decolorization efficiencies of crude laccase (1 U/mL) on α-NO at concentrations of 50, 100, 200, 400, 800, and 1000 mg/L were all around 90%. For MB13, the 24 h decolorization efficiency was 92% at 50 mg/L, and as the concentration increased, the efficiency gradually decreased, reaching 70% at 1000 mg/L.

**Figure 2:**
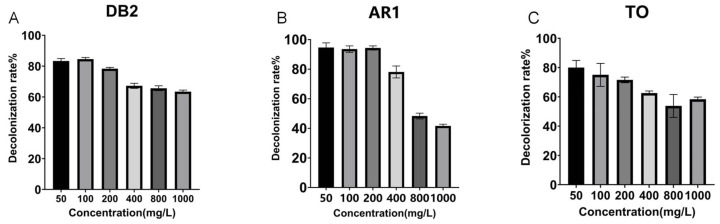
Decolorization of different azo dyes by crude laccase from the yang1 strain (2 U/mL) at 30 °C. (**A**). Decolorization efficiencies of different concentrations of azo dye DB2 treated with 2 U/mL crude laccase (24 h). (**B**). Decolorization efficiencies of different concentrations of azo dye AR1 treated with 2 U/mL crude laccase (24 h). (**C**). Decolorization efficiencies of different concentrations of azo dye TO treated with 2 U/mL crude laccase (24 h).

As illustrated in Figure 2, the 24 h decolorization efficiencies of crude laccase (2 U/mL) on TO and AR1 at each concentration were higher overall than those observed with 1 U/mL laccase. The above results showed that with the increase in laccase dosage, the decolorization efficiencies of azo dyes AR1 and TO were improved to a certain extent, especially for the dyes with a higher concentration.

#### 2.1.2. Time Course Decolorization of Different Concentrations of Azo Dyes by Crude Laccase from the Yang1 Strain

**Figure 3:**
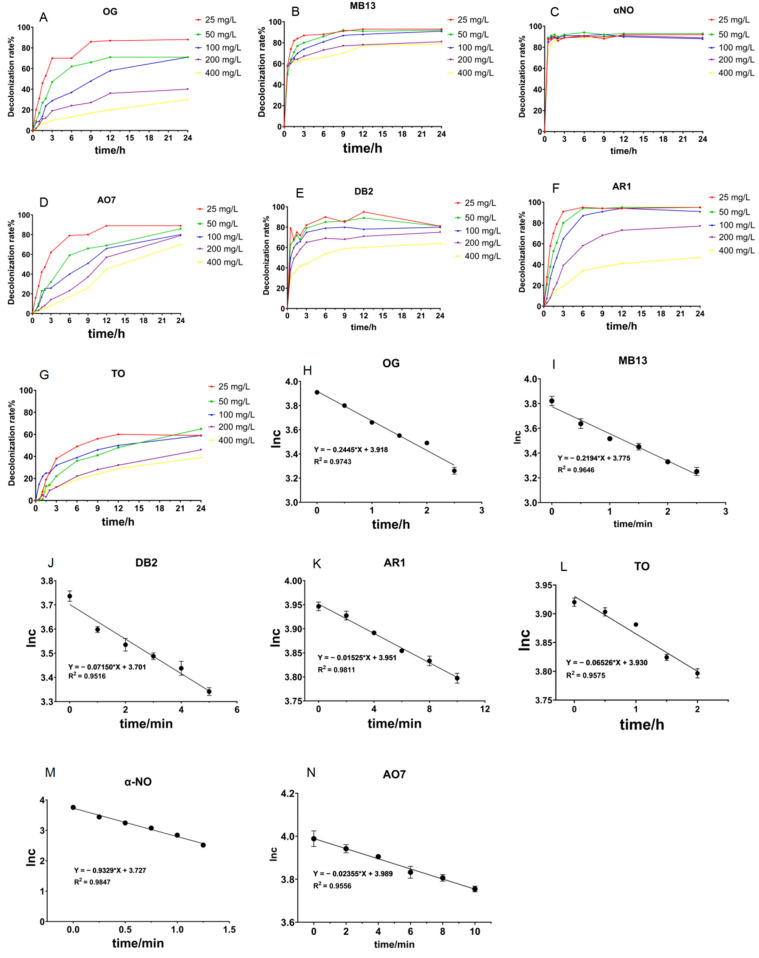
Time course decolorization of different azo dyes by crude laccase from the yang1 strain (1 U/mL) at 30 °C and ln c–t curves of the decolorization process of azo dyes. (**A**–**G**): Time course decolorization of different azo dyes by crude laccase ((**A**–**D**) at 1 U/mL, (**E**–**G**) at 2 U/mL). (**H**–**N**): ln c–t curves of the decolorization process of azo dyes by crude laccase. The unit of measurement for c is mg/L.

As illustrated in Figure 3A–G, when using 1 U/mL of crude laccase to decolorize various concentrations of OG, MB13, AO7, and α-NO across a time course, the majority of the decolorization of low dye concentrations (50, 100 mg/L) happened within 12 h. Crude laccase decolorized both α-NO and MB13 very quickly. It attained over 80% decolorization on α-NO at various concentrations in 0.5 h. Decolorization efficiencies of MB13 were above 60% in one hour, and the final decolorization efficiency was about 90%, achieving virtually full decolorization. Among the four azo dyes, crude laccase had the fastest rate of decolorization on α-NO. The order of the decolorization rates was α-NO > MB13 > AO7 > OG.

#### 2.1.3. Study on the Kinetic Equations of the Degradation of Different Azo Dyes by Crude Laccase from the Yang1 Strain

As illustrated in Figure 3H–K, the yang1 strain laccase’s degradation of seven azo dyes was well fitted to a straight line with R^2^ > 0.95. The above results indicated that the degradation of the seven azo dyes by crude laccase was a first-order reaction. The corresponding reaction rate constants for yang1 strain laccase’s degradation of α-NO, AO7, MB13, AR1, DB2, OG, and TO were 9.3 × 10^−1^ min^−1^, 2.4 × 10^−2^ min^−1^, 2.2 × 10^−1^ min^−1^, 1.5 × 10^−2^ min^−1^, 7.1 × 10^−2^ min^−1^, 2.4 × 10^−1^ h^−1^, and 6.5 × 10^−2^ h^−1^, respectively. The above results indicated that the reaction rate of crude laccase catalyzing the decolorization of seven azo dyes was ranked as follows: α-NO > MB13 > DB2 > AO7 > AR1 > OG > TO. The results of the degradation kinetics were consistent with the time course degradation results.

### 2.2. Study on Decolorization of Different Triphenylmethane Dyes by Crude Laccase from the Yang1 Strain

#### 2.2.1. Measurement of the 24 H Decolorization Efficiencies of Different Triphenylmethane Dyes Using Crude Laccase

**Figure 4:**
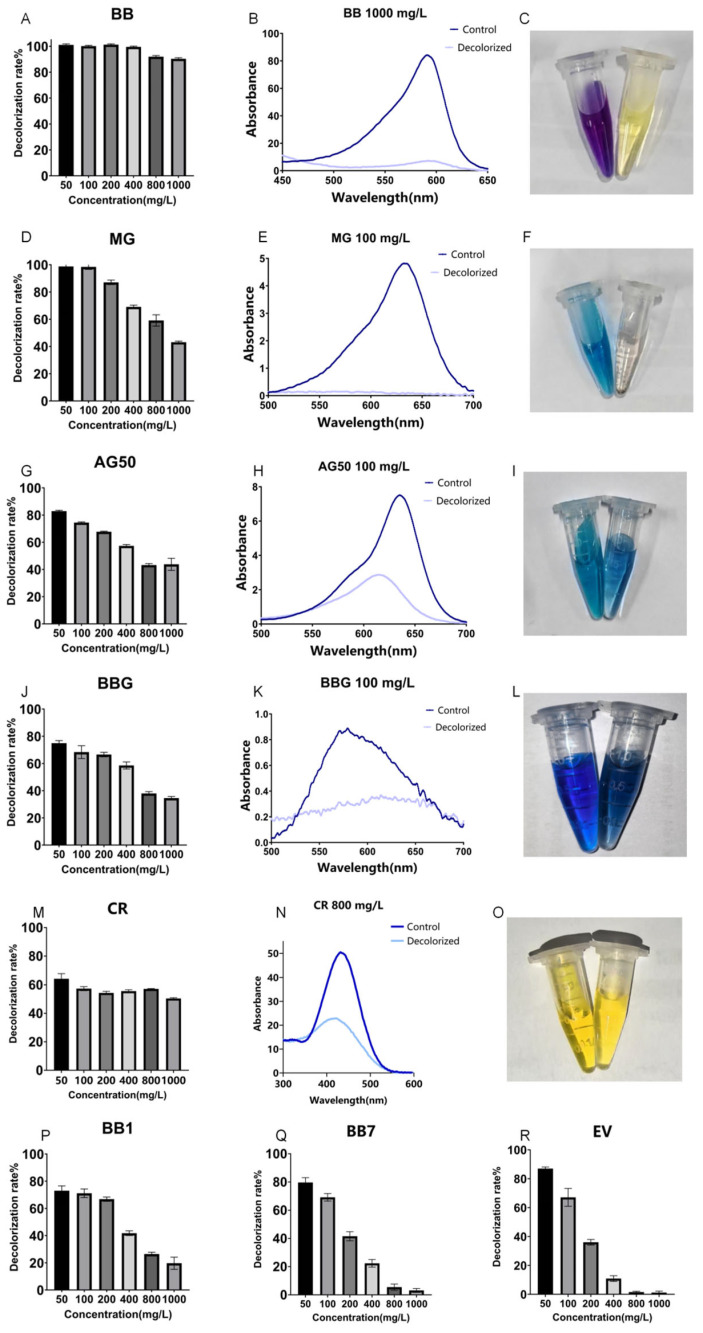
Decolorization of different triphenylmethane dyes by crude laccase from the yang1 strain at 30 °C. (**A**,**D**,**G**,**J**,**M**,**P**,**Q**,**R**): Decolorization efficiencies of different concentrations of triphenylmethane dyes BB, MG, AG50, BBG, CR, BB1, BB7, and EV treated with 1 U/mL crude laccase (24 h). (**B**,**E**,**H**,**K**,**N**): Spectral scanning graph of triphenylmethane dyes BB, MG, AG50, BBG, and CR before and after treatment with 1 U/mL crude laccase. **(C,F,I,L,O**): Color change diagram of the BB, MG, AG50, BBG, and CR dyes before and after laccase decolorization (left—before decolorization, right—after decolorization).

As shown in Figure 4, the 24 h decolorization efficiencies of crude laccase (1 U/mL) on 50, 100, 200, 400, 800, and 1000 mg/L BB were all above 90%. For MG, the 24 h decolorization efficiency was 99% at 50 mg/L, and reached 59% at 800 mg/L. According to the results described above, crude laccase had an excellent decolorization effect on BB and MG, a good decolorization effect on AG50, BBG, and CR, and a certain decolorization effect on BB7, EV, and BB1. Decolorization efficiencies for BB7 and EV dropped significantly as the concentration of the dye increased.

#### 2.2.2. Time Course Decolorization of Different Concentrations of Triphenylmethane Dyes by Crude Laccase from the Yang1 Strain

**Figure 5:**
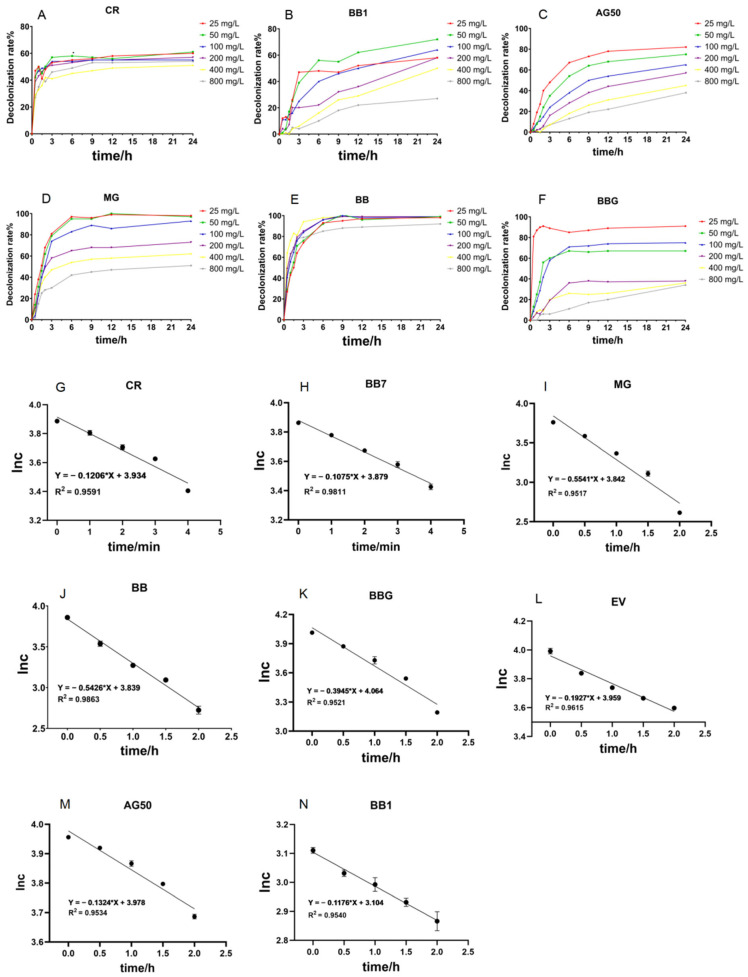
Time course decolorization of different triphenylmethane dyes by crude laccase from the yang1 strain (1 U/mL) at 30 °C and ln c–t curves of the decolorization process of triphenylmethane dyes. (**A**–**F**): Time course decolorization of different triphenylmethane dyes by crude laccase (1 U/mL). (**G**–**N**): ln c–t curves of the decolorization process of triphenylmethane dyes by crude laccase. The unit of measurement for c is mg/L.

As illustrated in Figure 5A–F, crude laccase was able to decolorize CR, BB, MG, and BB7 quickly, as demonstrated by the fast decolorization rate in the first three hours. Crude laccase had the fastest decolorization rate of BB and CR, with BB reaching more than 70% in 3 h. The decolorization rates of the BB1, BBG, AG50, MG, and EV dyes decreased by varying degrees as the concentration of the dye increased.

#### 2.2.3. Study on the Kinetic Equations of the Degradation of Different Triphenylmethane Dyes by Crude Laccase from the Yang1 Strain

As illustrated in Figure 5G–N, the degradation of eight triphenylmethane dyes by yang1 strain laccase was fitted as a straight line with R^2^ > 0.95, indicating a strong fitting relationship. This indicated that the degradation of the eight triphenylmethane dyes by crude laccase from the yang1 strain was a first-order reaction. The corresponding reaction rate constants for yang1 strain laccase’s degradation of CR, BB, BB1, BBG, AG50, MG, EV, and BB7 were 1.2 × 10^−1^ min^−1^, 5.4 × 10^−1^ h^−1^, 1.2 × 10^−1^ h^−1^, 3.9 × 10^−1^ h^−1^, 1.3 × 10^−1^ h^−1^, 5.5 × 10^−1^ h^−1^, 1.9 × 10^−1^ h^−1^, and 1.1 × 10^−1^ min^−1^, respectively. The above results indicated that the reaction rate of crude laccase catalyzing the decolorization of eight triphenylmethane dyes was ranked as follows: CR > BB7 > MG > BB > BBG > EV > AG50 > BB1.

### 2.3. Decolorization of Azo Dyes and Triphenylmethane Dyes in Successive Batches by Crude Laccase from the Yang1 Strain

In order to assess the stability and reusability of laccase in the consecutive decolorization process, as well as its capacity to decolorize different azo and triphenylmethane dyes in successive batches, we also conducted a study on the consecutive batch decolorization of azo and triphenylmethane dyes using crude laccase.

**Figure 6:**
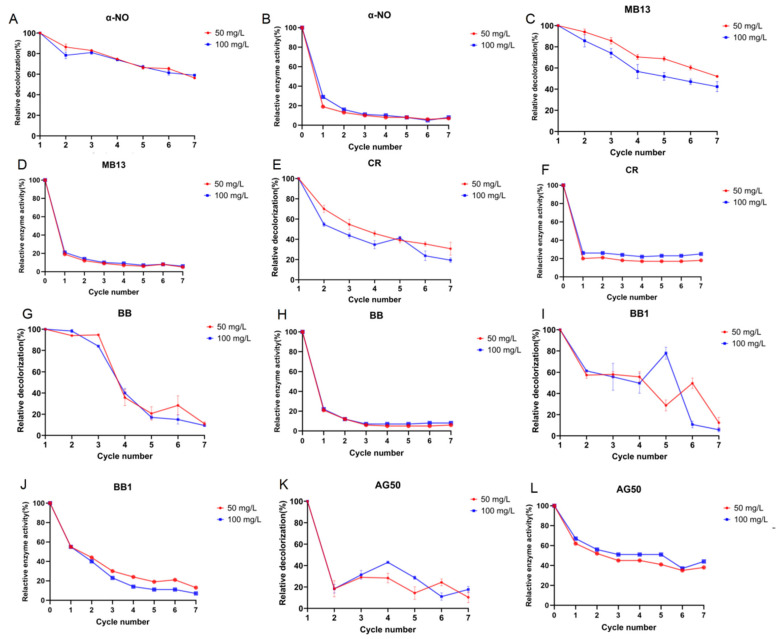
Continuous batch decolorization of various azo and triphenylmethane dyes using crude laccase at 30 °C. (**A**): The relative decolorization efficiency of crude laccase for azo dye α-NO over 7 consecutive 24 h batch decolorization rounds. (**B**): The relative laccase activity of crude laccase in the decolorization of azo dye α-NO across 7 consecutive 24 h batches. (**C**): The relative decolorization efficiency of crude laccase for azo dye MB13 over 7 consecutive 24 h batch decolorization rounds. (**D**): The relative laccase activity of crude laccase in the decolorization of azo dye MB13 across 7 consecutive 24 h batches. (**E**): The relative decolorization efficiency of crude laccase for triphenylmethane dye CR over 7 consecutive 24 h batch decolorization rounds. (**F**): The relative laccase activity of crude laccase in the decolorization of triphenylmethane dye CR across 7 consecutive 24 h batches. (**G**): The relative decolorization efficiency of crude laccase for triphenylmethane dye BB over 7 consecutive 24 h batch decolorization rounds. (**H**): The relative laccase activity of crude laccase in the decolorization of triphenylmethane dye BB across 7 consecutive 24 h batches. (**I**): The relative decolorization efficiency of crude laccase for triphenylmethane dye BB1 over 7 consecutive 24 h batch decolorization rounds. (**J**): The relative laccase activity of crude laccase in the decolorization of triphenylmethane dye BB1 across 7 consecutive 24 h batches. (**K**): The relative decolorization efficiency of crude laccase for triphenylmethane dye AG50 over 7 consecutive 24 h batch decolorization rounds. (**L**): The relative laccase activity of crude laccase in the decolorization of triphenylmethane dye AG50 across 7 consecutive 24 h batches.

As shown in Figure 6A,B, the relative decolorization efficiencies of 1 U/mL crude laccase against 50 mg/L of α-NO in seven consecutive rounds of decolorization ranged from 100% to 56%. The relative laccase activity after the seven decolorization cycles ranged from 19% to 7%. Additionally, 1 U/mL crude laccase also exhibited strong decolorization capability in continuous batches against 100 mg/L of α-NO.

As shown in Figure 6C, the relative decolorization efficiencies of 1 U/mL crude laccase against 50 mg/L of MB13 during seven successive rounds of decolorization ranged from 100% to 52%. The relative laccase activity after the seven decolorization reactions ranged from 19% to 5%. The relative decolorization efficiency of successive batches of azo dye MB13 decreased slightly, but it remained over 50% in the seventh round of the decolorization. The above results indicated that crude laccase from the yang1 strain had a strong ability to decolorize the azo dyes α-NO and MB13 in successive batches.

As shown in Figure 6E–L, the research results indicated that crude laccase from the yang1 strain had a good ability to decolorize the triphenylmethane dyes CR, BB, BB1, and AG50 in successive batches. The laccase activity maintained a good stability during the successive decolorization of CR and AG50.

### 2.4. Detoxification of Phytotoxicity of Various Azo Dyes by Crude Laccase

The above investigations revealed that crude laccase from the yang1 strain effectively decolorized azo and triphenylmethane dyes. To further investigate the detoxification effect of crude laccase on azo and triphenylmethane dyes, we examined the phytotoxicity changes in the dyes before and after laccase decolorization, using rice and wheat seed germination as test indicators.

**Figure 7:**
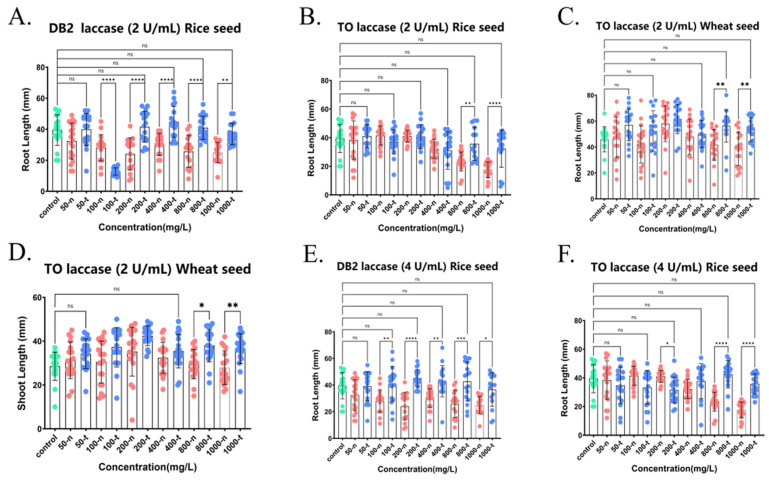
Detoxification of phytotoxicity of various azo dyes, DB2 and TO, by crude laccase (2 U/mL, 4 U/mL). (**A**). Effects of DB2 not treated by 2 U/mL crude laccase and DB2 treated by 2 U/mL crude laccase on the root growth of rice seeds. (**B**). Effects of TO not treated by 2 U/mL crude laccase and TO treated by 2 U/mL crude laccase on the root growth of rice seeds. (**C**). Effects of TO not treated by 2 U/mL crude laccase and TO treated by 2 U/mL crude laccase on the root growth of wheat seeds. (**D**). Effects of TO not treated by 2 U/mL crude laccase and TO treated by 2 U/mL crude laccase on the shoot growth of wheat seeds. (**E**). Effects of DB2 not treated by 4 U/mL crude laccase and DB2 treated by 4 U/mL crude laccase on the root growth of rice seeds. (**F**). Effects of TO not treated by 4 U/mL crude laccase and TO treated by 4 U/mL crude laccase on the root growth of rice seeds. ns meant no significant difference between groups, *p* > 0.05; * meant a significant difference between groups, *p* ≤ 0.05; ** meant a highly significant difference between groups, *p* ≤ 0.01; *** meant an extremely significant difference between groups, *p* ≤ 0.001; and **** meant an exceedingly significant difference between groups, *p* ≤ 0.0001. Green dots represents the blank control group. Red dots represents the control group (with dye, no crude laccase). Blue dots represents the experimental group (with both dye and crude laccase).

Figure 7A,E demonstrate the impact of azo dye DB2 on the growth of rice seed roots, both before and after decolorization by laccase at concentrations of 2 U/mL and 4 U/mL, respectively. Figure 7A showed that varied doses of DB2 not treated by laccase (200-n, 400-n, 800-n, and 100-n, where n means not treated by laccase) significantly inhibited the root growth of rice seeds, demonstrating that DB2 without laccase treatment was very toxic to rice seed root growth. However, laccase treatment significantly improved the root growth of rice seeds in DB2. The root lengths of rice seed in 200, 400, 600, and 800 mg/L DB2 (200-t, 400-t, 800-t, and 1000-t, where t means treated by laccase) treated with crude laccase (2 U/mL) were 1.73, 1.48, 1.59, and 1.48 times greater than those before laccase treatment (200-n, 400-n, 800-n, and 1000-n), respectively (*p* < 0.01). There was no significant difference (*p* > 0.05) in root length between the laccase-treated group (200-t, 400-t, 800-t, and 1000-t) and the blank control group (0 mg/L, control). The aforementioned results showed that the treatment with crude laccase (2 U/mL) totally eliminated the toxicity of 200, 400, 800, and 1000 mg/L DB2 on rice seed root growth (Figure 7A). As shown in Figure 7E, the root lengths of rice seed in 100, 200, 400, 800, and 1000 mg/L DB2 (100-t, 200-t, 400-t, 800-t, and 1000-t) treated with crude laccase (4 U/mL) were 1.32, 1.88, 1.42, 1.66, and 1.47 times greater than those before laccase treatment (100-n, 200-n, 400-n, 800-n, and 1000-n), respectively (*p* < 0.01). In addition, there was no significant difference (*p* > 0.05) in root length between the laccase-treated group (100-t, 200-t, 400-t, 800-t, and 1000-t) and the blank control group (0 mg/L, control). The aforementioned results indicated that the treatment with crude laccase (4 U/mL) totally eliminated the toxicity of 100, 200, 400, 800, and 1000 mg/L DB2 on rice seed root growth (Figure 7E). In conclusion, the decolorization by crude laccase had a very good detoxification effect on the toxicity of azo dye DB2 on rice seed root growth.

Figure 7B,F demonstrate the impact of azo dye TO on the growth of rice seed roots, both before and after decolorization by laccase at concentrations of 2 U/mL and 4 U/mL, respectively. Figure 7B shows that different concentrations of TO not treated by laccase (800-n and 1000-n) significantly inhibited the root growth of rice seeds. The results indicated that TO, without laccase treatment, exhibited strong toxicity towards the root growth of rice seeds. However, the root lengths of rice seed in 800 and 1000 mg/L TO (800-t and 1000-t) treated with crude laccase (2 U/mL) were 1.48 and 1.88 times greater than those before laccase treatment (800-n and 1000-n) (*p* < 0.01). Additionally, there was no significant difference (*p* > 0.05) in root length between the laccase-treated group (800-t and 1000-t) and the blank control group (0 mg/L, control). The aforementioned results showed that the treatment with crude laccase (2 U/mL) totally eliminated the toxicity of 800 and 1000 mg/L TO on rice seed root growth (Figure 7B). As shown in Figure 7F, the treatment of crude laccase (4 U/mL) also completely eliminated the toxicity of 800 and 1000 mg/L TO on the root growth of rice seeds. In conclusion, the decolorization of crude laccase from the yang1 strain also had a very good detoxification effect on the toxicity of another azo dye TO on the root growth of rice seeds.

Figure 7C,D show the effect of azo dye TO on the root and shoot growth of wheat seeds before and after decolorization with laccase (2 U/mL). The results showed that the treatment with crude laccase totally eliminated the toxicity of 800 and 1000 mg/L TO on wheat seed root growth. The treatment with laccase (2 U/mL) could also significantly reduce the toxicity of 800 and 1000 mg/L TO on the shoot growth of wheat seed.

In summary, the decolorization of crude laccase from the yang1 strain had a good detoxification effect on the toxicity of azo dyes DB2 and TO on wheat and rice seeds.

### 2.5. Detoxification of Phytotoxicity of Various Triphenylmethane Dyes by Crude Laccase

**Figure 8:**
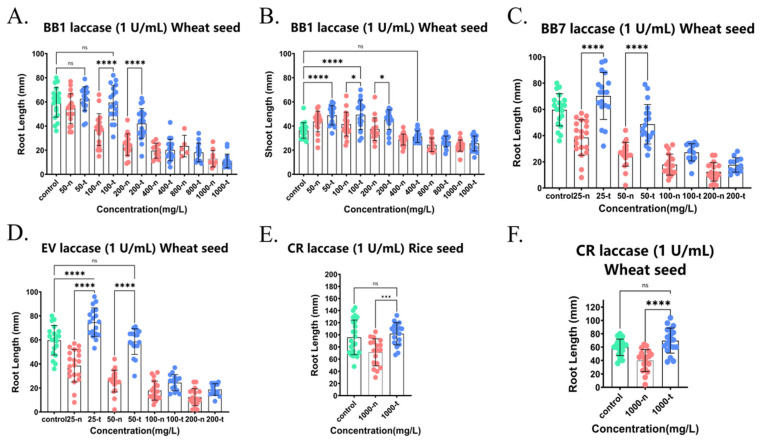
Detoxification of phytotoxicity of various triphenylmethane dyes BB1, BB7, EV, and CR by crude laccase (1 U/mL). (**A**). Effects of BB1 not treated by 1 U/mL crude laccase and BB1 treated by 1 U/mL crude laccase on the root growth of wheat seeds. (**B**). Effects of BB1 not treated by 1 U/mL crude laccase and BB1 treated by 1 U/mL crude laccase on the shoot growth of wheat seeds. (**C**). Effects of BB7 not treated by 1 U/mL crude laccase and BB7 treated by 1 U/mL crude laccase on the root growth of wheat seeds. (**D**). Effects of EV not treated by 1 U/mL crude laccase and EV treated by 1 U/mL crude laccase on the root growth of wheat seeds. (**E**). Effects of CR not treated by 1 U/mL crude laccase and CR treated by 1 U/mL crude laccase on the root growth of rice seeds. (**F**). Effects of CR not treated by 1 U/mL crude laccase and CR treated by 1 U/mL crude laccase on the root growth of wheat seeds. ns meant no significant difference between groups, *p* > 0.05; * meant a significant difference between groups, *p* ≤ 0.05; *** meant an extremely significant difference between groups, *p* ≤ 0.001; and **** meant an exceedingly significant difference between groups, *p* ≤ 0.0001. Green dots represents the blank control group. Red dots represents the control group (with dye, no crude laccase). Blue dots represents the experimental group (with both dye and crude laccase).

We also studied the detoxification effect of crude laccase from the yang1 strain on different triphenylmethane dyes. Figure 8A,B show the effect of triphenylmethane dye BB1 on the shoot growth and root growth of wheat seeds before and after decolorization with laccase (1 U/mL). As shown in Figure 8A, the root lengths of wheat seeds in 100 and 200 mg/L BB1 (100-t and 200-t) treated with crude laccase (1 U/mL) were 1.57 and 1.63 times greater than those before laccase treatment (100-n and 200-n) (*p* < 0.001). There was no significant difference (*p* > 0.05) in root length between the laccase-treated group (100-t) and the blank control group (0 mg/L, control). The aforementioned results showed that the treatment with crude laccase totally eliminated the toxicity of 100 mg/L BB1 on wheat seed root growth. Additionally, it can significantly reduce the toxicity of 200 mg/L BB1 on the root growth of wheat seeds.

Figure 8C–F show the effects of triphenylmethane dyes BB7, EV, and CR on rice seed root growth and wheat seed root growth before and after laccase decolorization. The results showed that crude laccase from the yang1 strain was able to significantly attenuate the toxicity of 25 and 50 mg/L BB7 on the root growth of wheat seed (Figure 8C). Crude laccase was able to completely eliminate the toxicity of 25, 50 mg/L EV on the root growth of wheat seed (Figure 8D). Crude laccase was able to completely eliminate the toxicity of 1000 mg/L CR on the root growth of rice and wheat seed (Figure 8E,F). In summary, the decolorization of crude laccase from the yang1 strain had a good detoxification effect on the toxicity of triphenylmethane dyes BB1, BB7, EV, and CR on both wheat and rice seeds.

### 2.6. Detoxification of Phytotoxicity of Two Dye Mixtures DB2 + BB7, DB2 + EV, TO + EV, DB2 + BB1, TO + BB1, TO + BB7, DB2 + EV (Azo Dye + Triphenylmethane Dye) by Crude Laccase

The composition of actual industrial dye wastewater is complex, often containing various dyes with distinct structures, which are discharged into the environment as mixed dyes. Therefore, systematically evaluating the detoxification capability of laccase towards mixed dyes containing different types of dyes plays a positive role and holds significant practical value in more effectively utilizing laccase to treat actual dye wastewater. The above results showed that crude laccase from the yang1 strain had a good detoxification effect on the phytotoxicity of single azo and triphenylmethane dyes. Does crude laccase have a good detoxification ability on a mixture of azo and triphenylmethane dyes (two types of dye mixtures)? To address this scientific question, we further explored the detoxification effect of crude laccase on a mixture of azo and triphenylmethane dyes.

**Figure 9:**
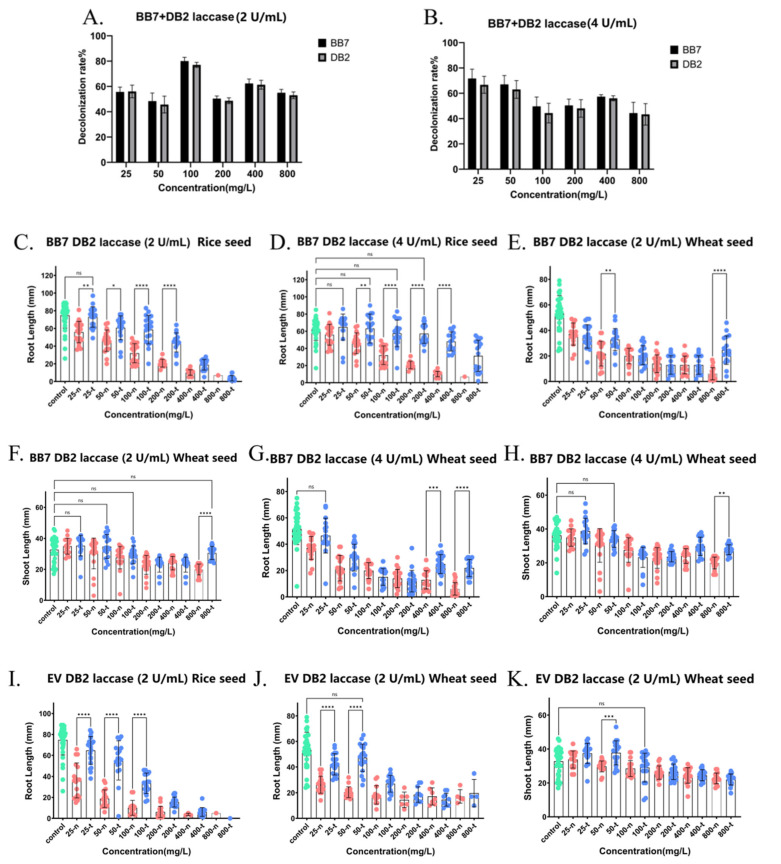

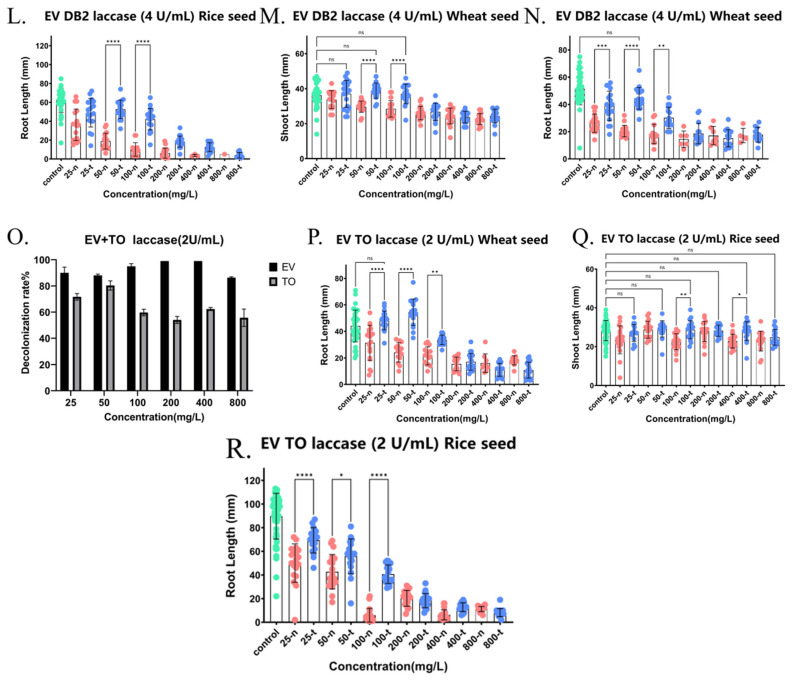
Detoxification of phytotoxicity of mixtures of two dyes (azo dye + triphenylmethane dye, BB7 + DB2, EV + DB2, EV + TO) by crude laccase. (**A**). 24 h decolorization efficiencies for BB7 + DB2 mixture at varying concentrations treated with 2 U/mL crude laccase. (**B**). 24 h decolorization efficiencies for BB7 + DB2 mixture at varying concentrations treated with 4 U/mL crude laccase. (**C**). Effects of BB7 + DB2 not treated by 2 U/mL crude laccase and BB7 + DB2 treated by 2 U/mL crude laccase on the root growth of rice seeds. (**D**). Effects of BB7 + DB2 not treated by 4 U/mL crude laccase and BB7 + DB2 treated by 4 U/mL crude laccase on the root growth of rice seeds. (**E**). Effects of BB7 + DB2 not treated by 2 U/mL crude laccase and BB7 + DB2 treated by 2 U/mL crude laccase on the root growth of wheat seeds. (**F**). Effects of BB7 + DB2 not treated by 2 U/mL crude laccase and BB7 + DB2 treated by 2 U/mL crude laccase on shoot growth of wheat seeds. (**G**). Effects of BB7 + DB2 not treated by 4 U/mL crude laccase and BB7 + DB2 treated by 4 U/mL crude laccase on root growth of wheat seeds. (**H**). Effects of BB7 + DB2 not treated by 4 U/mL crude laccase and BB7 + DB2 treated by 4 U/mL crude laccase on shoot growth of wheat seeds. (**I**). Effects of EV + DB2 not treated by 2 U/mL crude laccase and EV + DB2 treated by 2 U/mL crude laccase on root growth of rice seeds. (**J**). Effects of EV + DB2 not treated by 2 U/mL crude laccase and EV + DB2 treated by 2 U/mL crude laccase on root growth of wheat seeds. (**K**). Effects of EV + DB2 not treated by 2 U/mL crude laccase and EV + DB2 treated by 2 U/mL crude laccase on shoot growth of wheat seeds. (**L**). Effects of EV + DB2 not treated by 4 U/mL crude laccase and EV + DB2 treated by 4 U/mL crude laccase on root growth of rice seeds. (**M**). Effects of EV + DB2 not treated by 4 U/mL crude laccase and EV + DB2 treated by 4 U/mL crude laccase on shoot growth of wheat seeds. (**N**). Effects of EV + DB2 not treated by 4 U/mL crude laccase and EV + DB2 treated by 4 U/mL crude laccase on root growth of wheat seeds. (**O**). 24 h decolorization efficiencies for EV + TO mixture at varying concentrations treated with 2 U/mL crude laccase. (**P**). Effects of EV + TO not treated by 2 U/mL crude laccase and EV + TO treated by 2 U/mL crude laccase on root growth of wheat seeds. (**Q**). Effects of EV + TO not treated by 2 U/mL crude laccase and EV + TO treated by 2 U/mL crude laccase on shoot growth of rice seeds. (**R**). Effects of EV + TO not treated by 2 U/mL crude laccase and EV + TO treated by 2 U/mL crude laccase on root growth of rice seeds. xx-n: untreated by laccase. xx-t: treated by laccase. ns meant no significant difference between groups, *p* > 0.05; * meant a significant difference between groups, *p* ≤ 0.05; ** meant a highly significant difference between groups, *p* ≤ 0.01; *** meant an extremely significant difference between groups, *p* ≤ 0.001; and **** meant an exceedingly significant difference between groups, *p* ≤ 0.0001. Green dots represents the blank control group. Red dots represents the control group (with dye, no crude laccase). Blue dots represents the experimental group (with both dye and crude laccase).

As shown in Figure 9A,B, crude laccase (2 U/mL and 4 U/mL) demonstrated a good decolorization effect on various concentrations of DB2 + BB7 dye mixture. For instance, the 24 h decolorization efficiencies of DB2 and BB7 in a solution containing 100 + 100 mg/L of DB2 + BB7, treated with crude laccase at a concentration of 2 U/mL, were 80% and 77%, respectively. As shown in Figure 9C, after treatment with crude laccase (2 U/mL), the root lengths of rice seeds in 25 + 25, 50 + 50, 100 + 100, and 200 + 200 mg/L DB2 + BB7 (25-t, 50-t, 100-t, and 200-t) were found to be 1.31, 1.32, 1.83, and 2.14 times longer, respectively, compared with those before laccase treatment (25-n, 50-n, 100-n, and 200-n) (*p* < 0.01). There was no significant difference (*p* > 0.05) in root length between the laccase-treated group (25-t) and blank control group (0 mg/L). The above results indicated that the treatment of crude laccase (2 U/mL) was able to completely eliminate the toxicity of 25 + 25 mg/L DB2 + BB7 on the root growth of rice seeds, as well as significantly attenuate the toxicity of 50 + 50, 100 + 100, and 200 + 200 mg/L DB2 + BB7 on the root growth of rice seeds (Figure 9C). As shown in Figure 9D, after treatment with crude laccase (4 U/mL), the root lengths of rice seeds in 50 + 50, 100 + 100, 200 + 200, and 400 + 400 mg/L DB2 + BB7 (50-t, 100-t, 200-t, and 400-t) were found to be 1.37, 1.79, 2.77, and 4.74 times longer, respectively, compared with those before laccase treatment (50-n, 100-n, 200-n, and 400-n), respectively (*p* < 0.001). There was no significant difference (*p* > 0.05) in root length between the laccase-treated groups (50-t, 100-t, 200-t) and the blank control group (0 mg/L, control). The above results showed that the treatment of crude laccase (4 U/mL) was able to completely eliminate the toxicity of DB2 + BB7 on the root growth of rice seeds at concentrations of 50 + 50, 100 + 100, and 200 + 200 mg/L, as well as significantly reduce the toxicity of DB2 + BB7 at higher concentrations (400 + 400 and 800 + 800 mg/L) (Figure 9D). In summary, the laccase treatment showed a good detoxifying effect on the toxicity of DB2 + BB7 dye mixture on the root growth of rice seed. As shown in Figure 9E–H, the treatment of crude laccase (2 U/mL and 4 U/mL) could significantly attenuate or completely eliminate the toxicity of DB2 + BB7 mixed dyes on the shoot and root growth of wheat seed (Figure 9E–H). In conclusion, the treatment with crude laccase exhibited excellent detoxification effects on the toxicity of DB2 + BB7 mixed dye towards both wheat and rice seeds.

Figure 9I–N show the effects of the DB2 + EV dye mixture on the root and shoot growth of rice and wheat seeds before and after laccase decolorization. As shown in Figure 9I, the root lengths of rice seeds treated with crude laccase (25-t, 50-t, and 100-t) were 1.79, 2.93, and 3.32 times longer than those before treatment (25-n, 50-n, and 100-n), respectively (*p* < 0.0001). The above results showed that treating rice seeds with crude laccase (2 U/mL) could significantly attenuate the toxicity of 25 + 25, 50 + 50, and 100 + 100 mg/L DB2 + EV on the root growth. The treatment with crude laccase (2 U/mL) demonstrated good detoxification effects on the toxicity of DB2 + EV mixed dye towards rice seed root growth (Figure 9I). As shown in Figure 9J, the root lengths of wheat seed in 25 + 25 and 50 + 50 mg/L DB2 + EV (25-t and 50-t) treated with crude laccase (2 U/mL) were 1.60 and 2.31 times longer than those before laccase treatment (25-n and 50-n) (*p* < 0.0001). There was no significant difference (*p* > 0.05) in root length between the laccase-treated groups (25-t and 50-t) and blank control group (0 mg/L, control). The above results showed that treatment with crude laccase (2 U/mL) was able to totally remove the toxicity of 25 + 25 and 50 + 50 mg/L DB2 + EV dye mixture on wheat seed root growth (Figure 9J). As shown in Figure 9L–N, the treatment of crude laccase (4 U/mL) could also significantly reduce or completely eliminate the toxicity of DB2 + EV mixed dyes on the shoot and root growth of wheat seed and rice seed (Figure 9L–N). In summary, the treatment with crude laccase demonstrated excellent detoxification effects on the toxicity of DB2 + EV mixed dye towards both wheat and rice seeds.

Figure 9O–R show the effects of TO + EV dye mixtures before and after laccase decolorization on the root and shoot growth of rice and wheat seeds. As shown in Figure 9O, crude laccase (2 U/mL) showed good decolorization effect on different concentrations of TO + EV dye mixture. As shown in Figure 9P, the root lengths of wheat seeds treated with crude laccase (25-t, 50-t, and 100-t) were 1.39, 1.31, and 6.89 times longer than those before treatment (25-n, 50-n, and 100-n), respectively (*p* < 0.05). There was no significant change (*p* > 0.05) in root length between the laccase-treated groups (25-t) and the blank control group (0 mg/L, control). The above results showed that treating wheat seeds with crude laccase (2 U/mL) completely eliminated the toxicity of 25 + 25 mg/L TO + EV on wheat seed root growth, and it was also able to significantly attenuate the toxicity of higher concentrations (50 + 50 and 100 + 100 mg/L) of TO + EV on wheat seed root growth (Figure 9P). As shown in Figure 9Q, the treatment of crude laccase was able to completely eliminate the toxicity of 100 + 100 − 800 + 800 mg/L TO + EV on rice seed shoot growth (Figure 9Q). The treatment of crude laccase was able to significantly reduce the toxicity of 25 + 25, 50 + 50, and 100 + 100 mg/L TO + EV on rice seed root growth (Figure 9R). In summary, the treatment with crude laccase from the yang1 strain demonstrated excellent detoxification effects on the toxicity of rice and wheat seeds caused by different concentrations of TO + EV mixed dye.

**Figure 10:**
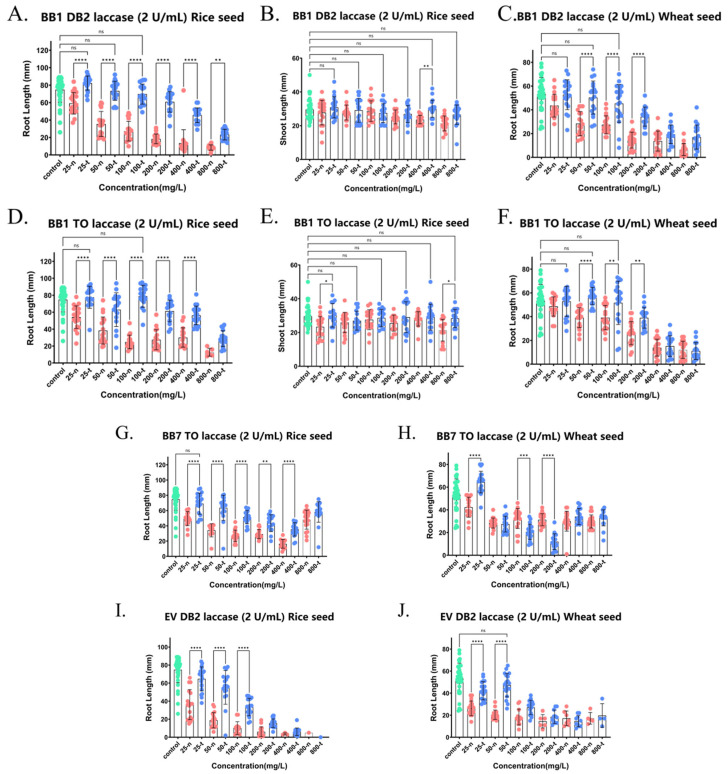
Detoxification of phytotoxicity of mixtures of two dye (azo dye + triphenylmethane dye, BB1 + DB2, BB1 + TO, BB7 + TO, EV + DB2) by crude laccase. (**A**). Effects of untreated BB1 + DB2 mixture and BB1 + DB2 mixture treated with 2 U/mL crude laccase on root growth of rice seeds. (**B**). Effects of untreated BB1 + DB2 mixture and BB1 + DB2 mixture treated with 2 U/mL crude laccase on shoot growth of rice seeds. (**C**). Effects of untreated BB1 + DB2 mixture and BB1 + DB2 mixture treated with 2 U/mL crude laccase extract on root growth of wheat seeds. (**D**). Effects of untreated BB1 + TO mixture and BB1 + TO mixture treated with 2 U/mL crude laccase on root growth of rice seeds. (**E**). Effects of untreated BB1 + TO mixture and BB1 + TO mixture treated with 2 U/mL crude laccase on shoot growth of rice seeds. (**F**). Effects of untreated BB1 + TO mixture and BB1 + TO mixture treated with 2 U/mL crude laccase on root growth of wheat seeds. (**G**). Effects of untreated BB7 + TO mixture and BB7 + TO mixture treated with 2 U/mL crude laccase extract on root growth of rice seeds. (**H**). Effects of untreated BB7 + TO mixture and BB7 + TO mixture treated with 2 U/mL crude laccase on root growth of wheat seeds. (**I**). Effects of untreated EV + DB2 mixture and EV + DB2 mixture treated with 2 U/mL crude laccase on root growth of rice seeds. (**J**). Effects of untreated EV + DB2 mixture and EV + DB2 mixture treated with 2 U/mL crude laccase on root growth of wheat seeds. xx-n: untreated by laccase. xx-t: treated by laccase. ns meant no significant difference between groups, *p* > 0.05; * meant a significant difference between groups, *p* ≤ 0.05; ** meant a highly significant difference between groups, *p* ≤ 0.01; *** meant an extremely significant difference between groups, *p* ≤ 0.001; and **** meant an exceedingly significant difference between groups, *p* ≤ 0.0001. Green dots represents the blank control group. Red dots represents the control group (with dye, no crude laccase). Blue dots represents the experimental group (with both dye and crude laccase).

Figure 10 shows the effects of mixtures of two dyes, DB2 + BB1, TO + BB1, TO + BB7, and DB2 + EV, before and after laccase decolorization on the root and shoot growth of rice and wheat seeds. The results indicated that the decolorization by laccase could significantly reduce or even completely eliminate the strong toxicity of DB2 + BB1, TO + BB1, TO + BB7, and DB2 + EV on the shoot and root growth of rice and wheat seeds. The decolorization by laccase exhibited excellent detoxification efficacy against the toxicity of mixed dyes DB2 + BB1, TO + BB1, TO + BB7, and DB2 + EV towards rice and wheat seeds.

### 2.7. Molecular Docking Studies of Yang1 Strain Laccase, LAC-Yang1, and Azo and Triphenylmethane Dyes

The above research results indicated that crude laccase from the yang1 strain exhibited excellent decolorization and detoxification effects on both single azo and triphenylmethane dyes, as well as mixtures of these two types of dyes. Preliminary research conducted in our laboratory has shown that crude laccase from the yang1 strain contained only one type of laccase enzyme, named LAC-Yang1 [26]. Furthermore, we have obtained the amino acid sequence of LAC-Yang1 laccase in our previous studies. Therefore, although we used crude laccase in this study to degrade and detoxify dyes, there was only one type of laccase, LAC-Yang1, present in the crude laccase solution. We believe that LAC-Yang1 laccase plays a major role in the decolorization and detoxification of dyes with different structural types by crude laccase. Thus, to better reveal the decolorization mechanism of crude laccase on dyes with different structures, we further conducted molecular docking studies between LAC-Yang1 laccase and various dyes. In order to verify the reliability and credibility of the molecular docking results and better reveal the differences in the binding mechanism between laccase and dyes, we used two types of molecular docking software.

#### 2.7.1. Molecular Docking of LAC-Yang1 Laccase and Azo Dyes Using Molegro Virtual Docker (MVD)

The results of the molecular docking of laccase and azo dyes are shown in Table 2. The MolDock score of the best binding conformation of the six azo dyes docked with laccase was less than −100. The MolDock score is the core scoring function of the MVD 2013.6.0.1 software, which is specifically utilized for screening optimal binding conformations and assessing the binding affinity of ligand molecules to receptor binding sites. The Re-Rank score is utilized to conduct a more precise re-evaluation and ranking of the ligand conformations obtained from preliminary docking, thereby enhancing the accuracy of the docking process. A lower scoring function indicates a lower binding energy, which signifies more stable binding between the ligand and the receptor protein. Binding affinity refers to the overall strength of non-covalent interactions between two molecules, with its essence primarily determined by intermolecular forces such as hydrogen bonding and van der Waals forces. The lower the binding affinity value, the tighter the binding. Therefore, the low binding affinity value of MB13 indicates its tight binding with the laccase protein, resulting in the high decolorization efficiency of laccase towards MB13 dye. This is consistent with the fact that the decolorization efficiency of laccase towards MB13 exceeds 90% (Table 2). The MolDock score and Re-Rank score of TO were large and the hydrogen bond binding energy (HBond) with laccase was almost zero, which indicated that TO had poor conformational stability for protein binding. This was consistent with the results of the previous experiments on the decolorization of different azo dyes by laccase, where it was known that the decolorization efficiencies of different concentrations of AO7, AR1, DB2, MB13, and α-NO were higher than 60%. However, the decolorization efficiency of TO was significantly lower than that of the other dyes, with a decolorization efficiency of less than 50%. It indicated that the stability of the binding conformation between laccase and dyes had an important impact on the decolorization effect of laccase on dyes.

**Figure 11:**
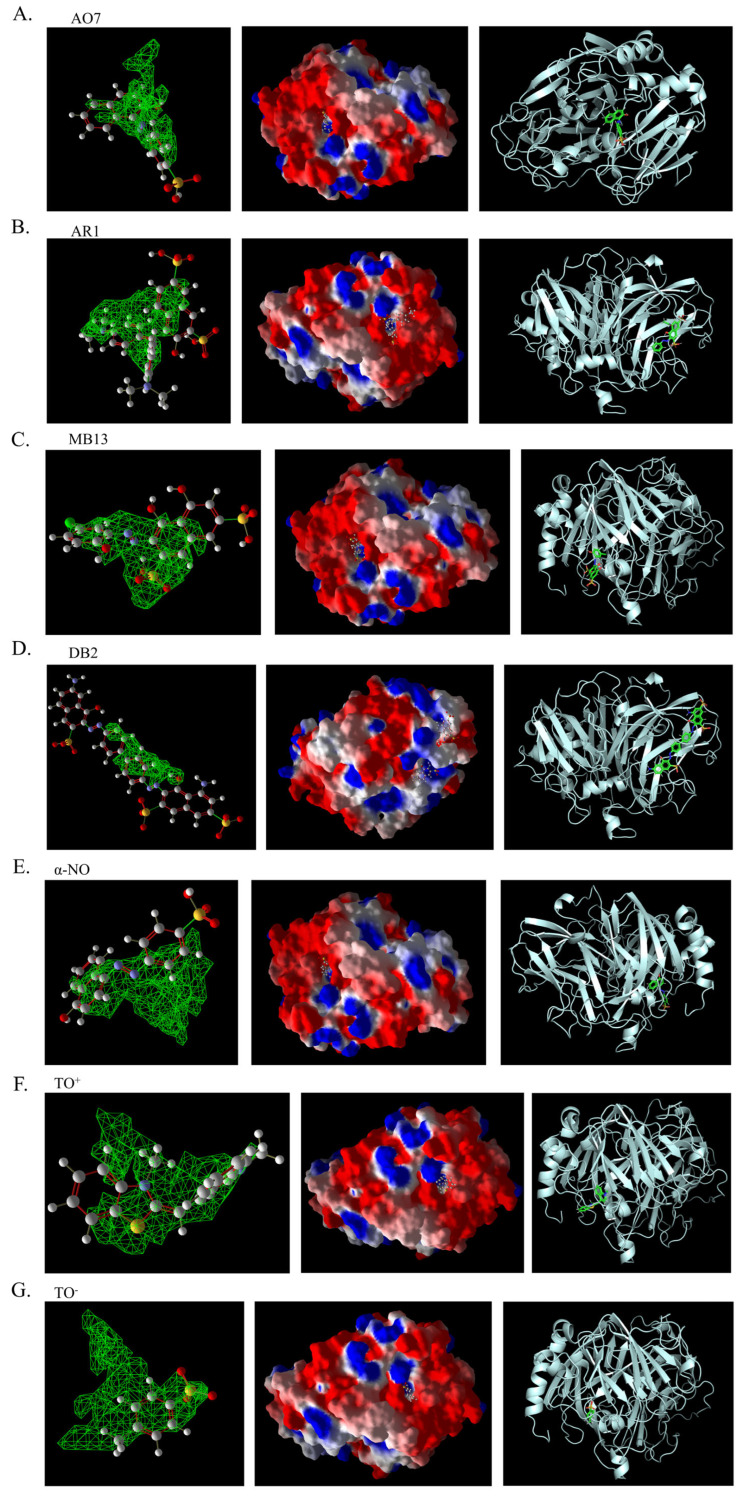
Three-dimensional structural images of molecular docking of different azo dyes and laccase using Molegro Virtual Docker (MVD). The first column is the 3D binding image of the dye ligand to the predicted binding pocket generated by MVD; the second column is the 3D binding image of the dye to the electrostatic surface of laccase generated by MVD; and the third column is the 3D binding image of the dye to laccase generated by PyMOL. (**A**). AO7. (**B**). AR1. (**C**). MB13. (**D**) DB2. (**E**). α-NO. (**F**). TO+. (**G**). TO-.

**Figure 12:**
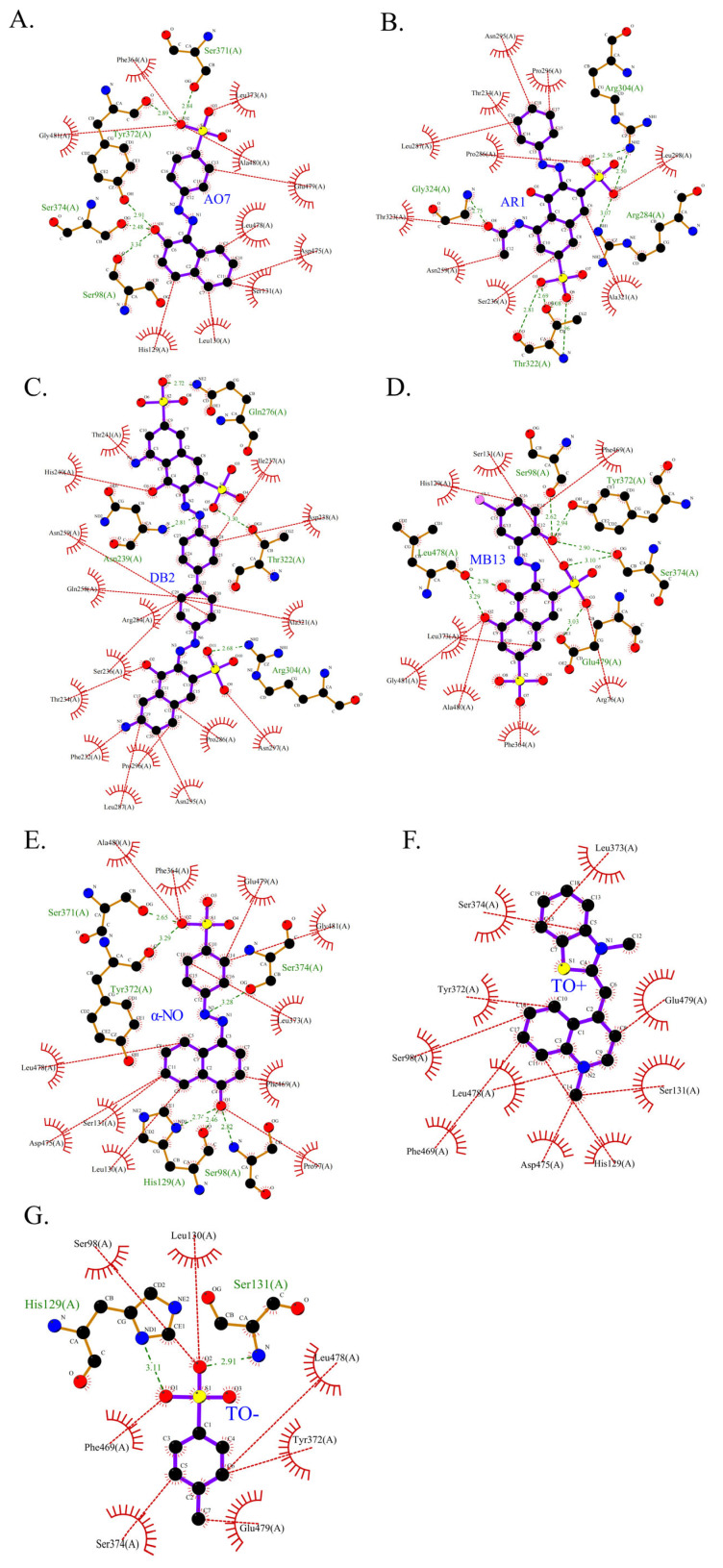
Two-dimensional structure of molecular docking of different azo dyes and laccase. The purple stick model represents the ligand, the orange stick model represents the protein structure, the red dashed line represents representative hydrophobic interactions, the red serrated semicircle represents amino acid residues that have hydrophobic interactions with the ligand, and the green dashed line represents hydrogen bonds. (**A**–**G**). (**A**). AO7. (**B**). AR1. (**C**). DB2. (**D**). MB13. (**E**). α-NO. (**F**). TO+. (**G**). TO-.

As shown in Table 3, the hydrophobic interactions and the number of hydrogen bonds between the three dyes, DB2, MB13, and α-NO, and the laccase were all higher, and the hydrogen bond lengths were short, mostly in the range of 2.5 Å–3 Å (Figure 11). The shorter the hydrogen bond bond length, the stronger the interaction. It indicated a better match with laccase and a better stability of the laccase–dye complex. It coincided with the results of the decolorization experiments, where the laccase was more effective in decolorizing the three dyes DB2, MB13 and α-NO. However, the cations and anions of TO had relatively few hydrophobic interactions with laccase, with 39 and 21 interactions, respectively. The number of hydrogen bonds between the cation and anion of TO and the laccase was 0 and 2, which is small in number (Table 3, Figure 12F,G). It indicated a poor match with laccase. The results of the decolorization experiments also showed that laccase was less effective in decolorizing TO, and the molecular docking results explained the possible reasons and mechanisms behind this phenomenon.

**Figure 13:**
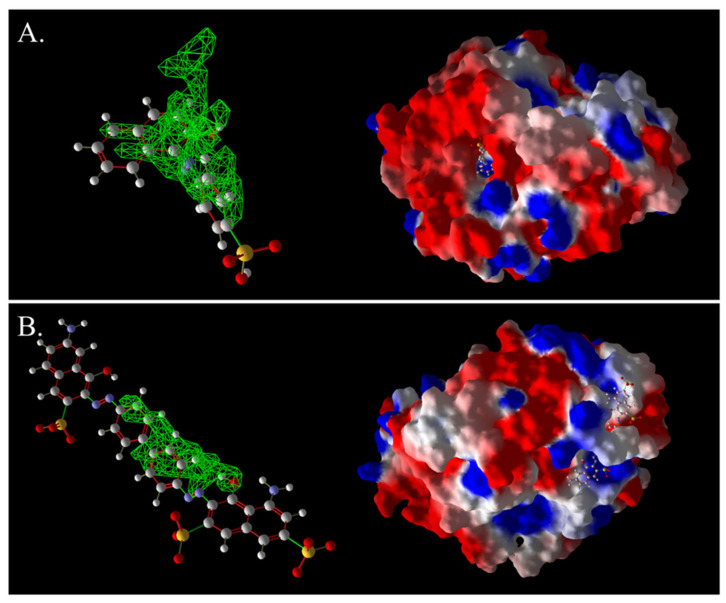
Three-dimensional images of AO7 and laccase complexes (**A**) and 3D images of DB2 and laccase complexes (**B**). The green grid represents a predictive docking pocket.

As shown in Figure 13A, AO7 was encapsulated in the predicted docking pocket (the part shown in the green grid), which could enter the catalytic pocket of laccase better and thus was easily degraded (Figure 13A). On the contrary, DB2, with a larger molecular weight and an elongated molecular structure, had most of its structure exposed outside the predicted binding pocket and did not enter into the catalytic region (Figure 13B). This result could explain the phenomenon that although DB2 had the lowest MolDock score and binding affinity, indicating that it binds most stably to laccase, the decolorization efficiency of DB2 significantly decreased as the dye concentration increased.

#### 2.7.2. Molecular Docking of LAC-Yang1 Laccase and Triphenylmethane Dye Using Molegro Virtual Docker (MVD)

**Figure 14:**
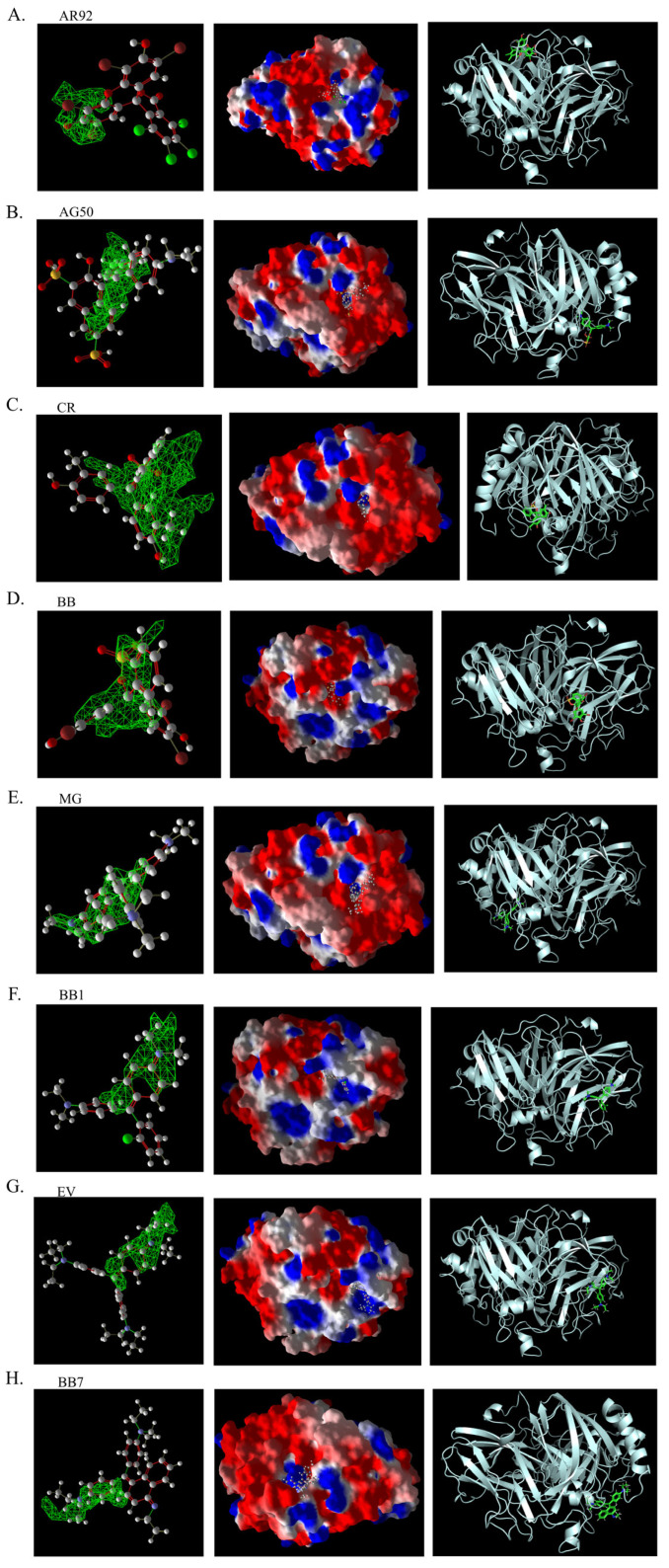
Three-dimensional structural images of molecular docking of different triphenylmethane dyes and laccase using Molegro Virtual Docker (MVD). The first column is the 3D binding image of the dye ligand to the predicted binding pocket generated by MVD; the second column is the 3D binding image of the dye to the electrostatic surface of laccase generated by MVD; and the third column is the 3D binding image of the dye to laccase generated by PyMOL. (**A**). AR92. (**B**). AG50. (**C**). CR. (**D**) BB. (**E**). MG. (**F**). BB1. (**G**). EV. (**H**). BB7.

**Figure 15:**
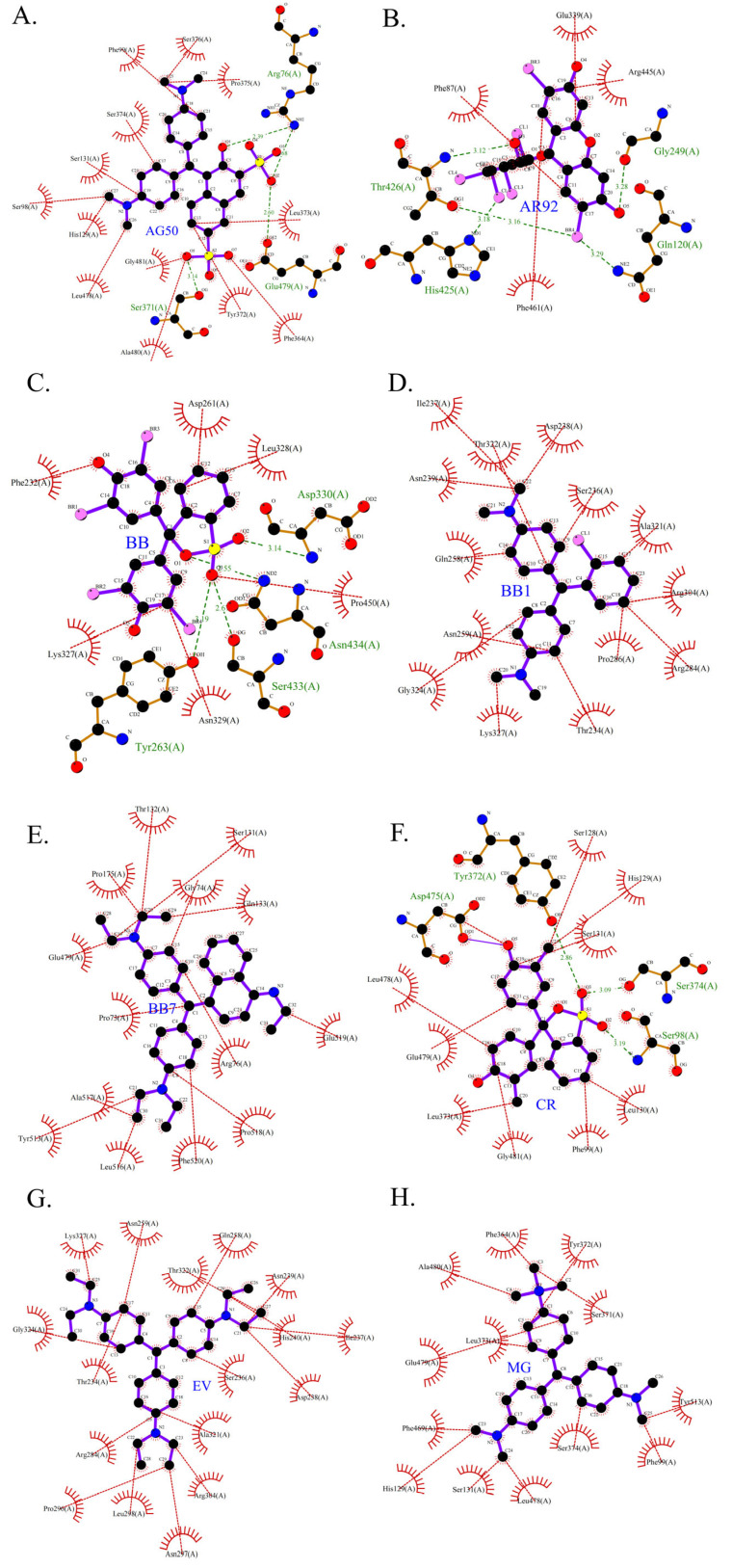
Two-dimensional structure of molecular docking of different triphenylmethane dyes and laccase. The purple stick model represents the ligand, the orange stick model represents the protein structure, the red dashed line represents representative hydrophobic interactions, the red serrated semicircle represents amino acid residues that have hydrophobic interactions with the ligand, and the green dashed line represents hydrogen bonds. (**A**). AG50. (**B**). AR92. (**C**). BB. (**D**). BB1. (**E**). BB7. (**F**). CR. (**G**). EV. (**H**). MG.

The 3D structural images of the molecular docking of different triphenylmethane dyes and laccase are shown in Figure 14. The two-dimensional structure of the molecular docking of different triphenylmethane dyes and laccase is shown in Figure 15.

**Figure 16:**
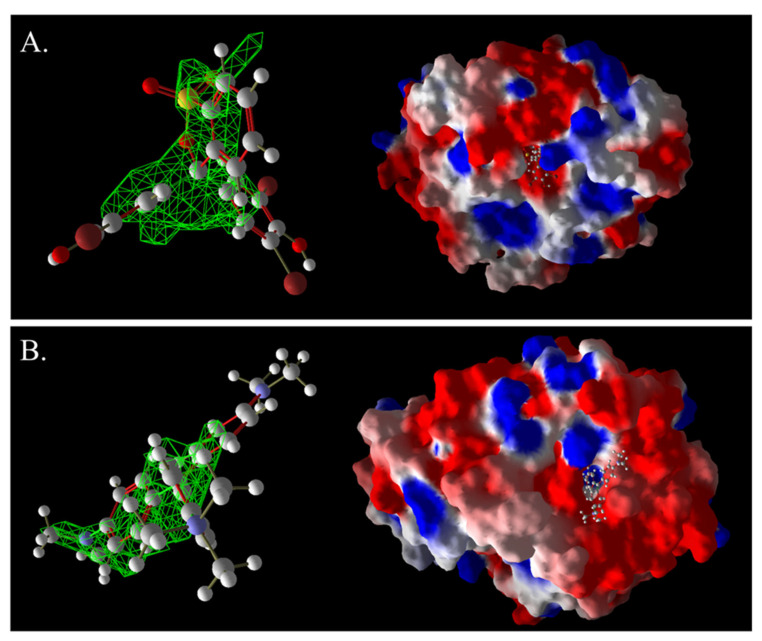
Three-dimensional images of BB and laccase complexes (**A**) and 3D images of MG and laccase complexes (**B**). The green grid represents a predictive docking pocket.

As shown in Table 4, the MolDock score for BB and laccase was relatively low, with a binding affinity of −38.4771 kJ/mol, indicating that BB bound tightly to laccase and formed a stable complex. As shown in Figure 16A, the molecular structure of BB was almost completely incorporated into the predicted laccase-binding pocket, further confirming the good binding between BB and laccase. This was consistent with the previous experimental results showing that laccase had a high decolorization rate for BB. MG also showed a high decolorization rate in the decolorization experiment, but its MolDock score for docking with laccase was higher than that of the other three triphenylmethane dyes, with a hydrogen bond energy of almost zero and a large binding affinity value. This indicated that its binding to the laccase protein was generally moderate, but as can be seen from Figure 16B, most of the molecular structure of MG was in the predicted laccase-binding pocket, which may be the reason for its high decolorization rate.

Additionally, as shown in Table 5, BB and MG exhibit multiple hydrophobic interactions with laccase. Among these, MG and laccase demonstrate 13 representative hydrophobic interactions. As illustrated in Figure 15, MG was connected to multiple amino acid residues (e.g., Glu479 (A), Leu373 (A), Ser374 (A), etc.), while BB was connected to multiple amino acid residues (such as Asp261 (A), Asn434 (A), Leu328 (A), etc.), indicating the presence of abundant binding sites. Charged amino acid residues such as aspartic acid (Asp) and glutamic acid (Glu) can form electrostatic attractions with oppositely charged regions of the dye molecules, thereby enhancing binding affinity. Laccase contained residues with polar groups, such as serine (Ser) and asparagine (Asn), which form multiple hydrogen bonds with BB. The average bond length of these hydrogen bonds was approximately 3 Å. Shorter bond lengths implied stronger forces, further enhancing the stability of the complex. The aforementioned molecular docking results elucidated the potential reasons and mechanisms underlying the remarkable decolorizing effect of laccase on BB and MG dyes.

**Figure 17:**
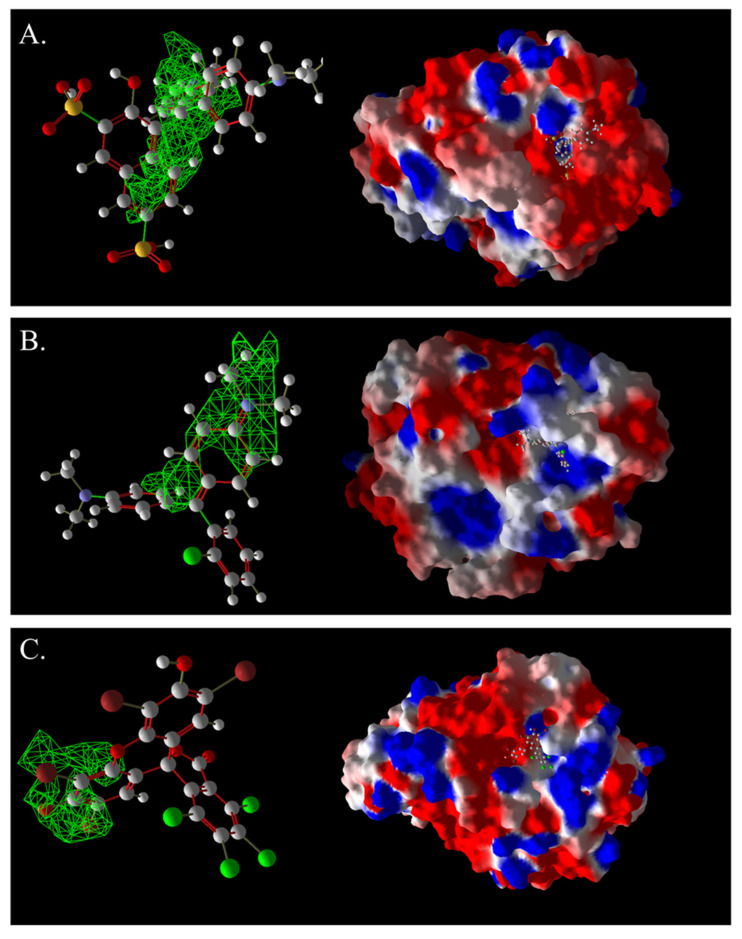
Three-dimensional images of AG50, BB7, AR92, and laccase complexes. (**A**): AG50; (**B**): BB7; (**C**): AR92. The green grid represents a predictive docking pocket.

The decolorization efficiencies of AG50, BB7, EV, and BB1 were approximately 80–60%, indicating good decolorization effects. AG50 and BB7 had low MolDock scores and Re-Rank scores for the best docking complex with laccase. BB7 binding affinity energy was low (Table 4). AG50 and BB7 also had more and diverse hydrophobic interactions with laccase, which can be seen in the better stability of the laccase and dye substrate complexes, thus improving the catalytic efficiency of laccase (Table 5). However, compared with MG and BB, AG50 and BB7 did not bind sufficiently to the predicted binding pocket of laccase (Figure 17). The results of this molecular docking were consistent with the findings of the laccase decolorization experiment, indicating that the decolorization efficiencies of laccase on AG50 and BB7 were lower than those on MG and BB.

The complex of AR92 with laccase had a low MolDock score, Re-Rank score, and binding affinity, and also had a high number and variety of hydrophobic interactions and hydrogen bonds. It indicated that the complex with the laccase protein was structurally stable. However, AR92 did not have a good binding orientation to the predicted binding pocket, and only a small fraction of AR92 was incorporated into the predicted binding pocket (Figure 17). Despite the structural stability of the formed complex, it did not enter the catalytic pocket well and the catalytic efficiency was low, which led to the poor decolorization of AR92 by laccase. The aforementioned molecular docking results elucidated the potential reasons and mechanisms behind the notably weak decolorizing effect of laccase on AR92 dye.

#### 2.7.3. Molecular Docking of LAC-Yang1 Laccase with Azo Dyes and Triphenylmethane Dyes Using AutoDock 4.2.6 Software

The docking results of various azo dyes and triphenylmethane dyes with LAC-Yang1 laccase were different. The binding energies of seven azo dye molecules docking with protein receptors were basically less than −16.75 kJ/mol. The dye molecules with a binding energy below −16.75 kJ/mol had medium or higher binding forces with laccase; the binding energy of MB13 to laccase was −16.71 kJ/mol, which was similar to −16.75 kJ/mol, and also had a good binding to laccase. The binding energies of the six triphenylmethane dye molecules docking with protein receptors were all less than −16.75 kJ/mol, and BB had the lowest binding energy, which meant that BB was the most tightly bound to laccase among these dye molecules. This was consistent with the previous results of the laccase decolorization of different concentrations of dye. The decolorization efficiency of BB can reach 100% at medium and high concentrations, indicating that laccase exhibited a highly effective decolorizing ability towards BB. The binding energy of BB7 dye docking with laccase was −23.49 kJ/mol, which was the highest among these dye molecules, and the inhibition constant of BB7 docking with laccase was 77.38 μmol/L, which was also the highest among these dye molecules. The above results indicated that among the six triphenylmethane dye molecules, the binding between the BB7 dye and the laccase protein was the least stable, and the degree of tightness was also relatively low. This result could also explain why the decolorization efficiency of BB7 dye by laccase was the lowest among the six triphenylmethane dyes. The results of the decolorization experiment showed that the decolorization efficiency of laccase on BB7 was only 30%, which was significantly lower than that on five other triphenylmethane dyes.

In azo dye molecules, the decolorization efficiencies of α-NO, AR1, and DB2 decreased as the dye concentration increased. This was speculated to be due to their larger molecular weights, which intensified intermolecular competition with increasing concentration, leading to unstable hydrophobic and hydrogen bonding interactions or the inhibition of the active sites of the laccase protein. The decolorization efficiencies of AO7, MB13, and OG were the highest at the middle concentration, which was related to the hydrophobic behavior and hydrogen bond strength between them and laccase. At low dye concentrations, laccase was not saturated, while at excessively high dye concentrations, its activity was inhibited. As the concentration of dye molecules continued to increase, there may be an inhibition of laccase activity, leading to a decrease in the decolorization efficiency.

The images generated by PyMOL 3.1.3.1 and LigPlot+ v.2.2.9 software showed that the azo dyes such as AO7, α-NO, and MB13 could enter the laccase pocket. Except DB2 and AR1, the key amino acid residues for the hydrophobic interaction of most azo dyes with laccase included Pro490, Arg489, Ile401, Pro403, Ala491, etc., and the key sites for hydrogen bonding were Ala491 and Arg388. Although their hydrophobic effects were weaker than that of triphenylmethane dyes, they formed more hydrogen bonds and exhibited more stable binding. BB dye can enter the catalytic pocket of laccase. Except for BB, the other triphenylmethane dyes formed no hydrogen bonds with the laccase protein. Their key hydrophobic interaction sites were different, and there was generally more hydrophobic interaction, facilitating tight binding and degradation. This result was consistent with the experimental result of high decolorization efficiency. However, the non-specificity of hydrophobic interaction may affect the recognition and catalysis of specific substrates by laccase, which explained the phenomenon that EV dye had the highest number of hydrophobic interactions but not the highest decolorization efficiency.

#### 2.7.4. Commonalities in Molecular Docking Results Obtained Using Two Different Types of Molecular Docking Software

We carried out molecular docking studies of laccase with azo and triphenylmethane dyes using two molecular docking softwares, MVD 2013.6.0.1 and AutoDock 4.2.6, respectively. The common results obtained by these two types of molecular docking software are described below: (1) The docking results of azo dyes α-NO, DB2, and MB13 with LAC-Yang1 laccase using MVD software showed that the scoring functions of these three azo dyes were all low, all below −115. The results analyzed by AutoDock software showed that the binding energies of these three azo dyes with laccase were also low, basically below −16.75 kJ/mol. Both of the above results indicated that the three azo dyes, α-NO, DB2 and MB13, had a better degree of binding with LAC-Yang1 laccase, and the binding force was also stronger. In addition, the results of the analysis from both types of software on the intermolecular forces between laccase and the dyes showed that the three azo dyes had more hydrogen bonds and hydrophobic interactions with LAC-Yang1 laccase, which also indicated that their binding with laccase was tight. The results of the previous decolorization experiments showed that the 24 h decolorization efficiencies of 10 mg/L of α-NO, DB2, and MB13 were above 90%, which basically achieved complete decolorization. Therefore, the molecular docking results obtained using two different software programs were consistent with the high decolorization efficiencies achieved for the α-NO, DB2, and MB13 azo dyes in the decolorization experiment. (2) The molecular docking studies of triphenylmethane dyes with laccase based on MVD and AutoDock showed that the scoring function and quadratic scoring function of MG and BB were low, and the binding energies were below −16.75 kJ/mol. Moreover, the two triphenylmethane dyes had multiple hydrophobic interactions with laccase, and both dye molecules were well incorporated into the predicted laccase-binding pocket. The results obtained by the two types of molecular docking software showed that MG and BB had a strong binding stability and a good binding ability with laccase. This was consistent with the results of MG and BB showing high decolorization efficiencies in the decolorization experiment.

#### 2.7.5. Summary of Molecular Docking Results Between Laccase and Azo Dyes and Triphenylmethane Dyes

In summary, the molecular docking studies revealed the interaction mechanism between laccase and dye molecules. Through molecular docking experiments using MVD and AutoDock software, it was found that the binding stability of dye and laccase (MolDock score, hydrogen bond binding energy, etc.) significantly affected the decolorization effect. Azo dyes such as AO7 and DB2 had high decolorization efficiencies (over 60%) due to their low binding energies, strong hydrogen bonds, and hydrophobic interactions. However, because of the poor conformational stability of TO, the decolorization efficiency of TO by laccase was lower (less than 50%). In triphenylmethane dyes, MG and BB were fully embedded in the laccase-binding pocket, leading to an excellent decolorization effect of laccase on these two dyes, with a decolorization efficiency exceeding 97%. MG and BB were electrostatically attracted to charged or polar amino acids, enhancing the binding affinity of the dye to laccase protein. The binding energies of α-NO and BB were −21.48 kJ/mol and −31.23 kJ/mol, respectively, both below −16.75 kJ/mol, indicating that they had a medium or higher binding power with laccase. Additionally, the molecular size of the dye and the degree to which it entered the catalytic pocket also affected the decolorization effect. For example, due to its large molecular size, DB2 dye cannot fully penetrate into the catalytic pocket, leading to a decrease in the decolorization efficiency of DB2 dye as its concentration increased. This study confirmed that molecular docking can explain the reasons and potential mechanisms behind the differences in catalytic abilities of laccase towards dyes with different structures from a structural perspective.

## 3. Discussion

### 3.1. Comparison of the Decolorization Ability Towards Two Types of Dyes of Crude Laccase from the Yang1 Strain with Those Reported in the Literature

#### 3.1.1. Azo Dyes

The study conducted by Bucchieri et al. revealed that, in the presence of the mediator ABTS, the 24 h decolorization efficiency of azo dye OG by laccase TP-LAC2 from *Trametes polyzona* reached 80% [27]. However, in this study, crude laccase from the yang1 strain achieved a decolorization efficiency of over 90% for the azo dye OG within 24 h in the absence of any mediator. Zeng et al. found that in the absence of any mediator, both crude laccase and purified laccase from *Trametes trogii* exhibited a decolorization efficiency of less than 10% for 10 mg/L azo dye AR1 in 3 h. However, upon the addition of the HOBT mediator, the decolorization efficiency could reach 90% [28]. In this study, crude laccase from the yang1 strain achieved a decolorization efficiency of 90% for 50 mg/L azo dye AR1 in 3 h without any mediator, demonstrating significant advantages in the decolorization ability. In summary, compared with the laccase reported in the literature [27,28], crude laccase from the yang1 strain demonstrated significant advantages in terms of its decolorizing ability towards azo dyes such as OG and AR1.

#### 3.1.2. Triphenylmethane Dyes

Sun et al. found that the decolorization efficiency of laccase from *Gymnopus luxurians* for the triphenylmethane dyes Methyl Green (MG) and Bromophenol Blue (BB) was over 80% within 24 h [29]. Grassi et al. found that the laccase from *Trametes trogii* also exhibited a decolorization efficiency of over 80% for MG and BB within 24 h [7]. In this study, crude laccase from the yang1 strain demonstrated a higher decolorization efficiency of over 90% for MG and BB within 24 h, achieving almost complete decolorization. Yang et al. found that a novel laccase, Lac1326, achieved a decolorization efficiency of only 22% for BB degradation in 12 h in the absence of a mediator, but the decolorization efficiency could reach 80% in 12 h in the presence of ABTS as a mediator [30]. However, in this study, crude laccase from the yang1 strain can achieve a decolorization efficiency of over 80% for triphenylmethane dye BB at concentrations ranging from 25 to 800 mg/L within 12 h, without the presence of a mediator. Yang et al. found that the decolorization efficiency of a novel laccase LacA at 2 U/mL was 57% for the triphenylmethane dye CR [31]. However, in this study, crude laccase from the yang1 strain achieved a decolorization efficiency of over 60% for the triphenylmethane dye CR at 1 U/mL. Therefore, compared with the laccase reported in the literature [7,29,30,31], crude laccase from the yang1 strain also demonstrated significant advantages in decolorizing triphenylmethane dyes.

### 3.2. The Research Significance and Practical Value of Crude Laccase from the Yang1 Strain, Which Exhibits Strong Decolorizing Ability Towards Mixed Dyes (Azo + Triphenylmethane)

In industrial production processes such as textiles, printing, and dyeing, the resulting dye wastewater often contains various types of dyes, among which azo dyes and triphenylmethane dyes are the most common types. Therefore, the decolorizing capability of laccase towards various dye mixtures is a crucial determinant of the effectiveness of laccase treatment in actual dye wastewater. Previous studies have primarily focused on the decolorization of individual dyes, whereas actual dye wastewater is complex and typically contains multiple different types of dyes. In this study, crude laccase from the yang1 strain demonstrated a strong decolorizing ability, not only towards single dyes, but also towards various mixed dyes in different combinations (azo + triphenylmethane). This result indicated that crude laccase from the yang1 strain had great potential and advantages in the efficient treatment of actual dye wastewater containing various types of dyes. The finding that crude laccase from the yang1 strain exhibits strong decolorizing capabilities for mixed dyes holds significant theoretical and practical value for its practical application in the efficient treatment of dye wastewater.

### 3.3. The Research Significance and Practical Value of Crude Laccase from the Yang1 Strain Exhibiting Strong Continuous Batch Decolorization Capability for Azo and Triphenylmethane Dyes

In this study, crude laccase from the yang1 strain exhibited a strong continuous batch decolorization ability towards azo dyes such as α-NO and MB13, as well as triphenylmethane dyes such as CR, BB, and BB1. Additionally, the laccase activity maintained good stability during the continuous decolorization process. This indicated that crude laccase from the yang1 strain exhibited a strong sustainable degradation capability, and laccase demonstrated good reusability. In the practical application of treating dye wastewater with laccase, the sustainable degradation capability and reusability of laccase are key factors in reducing treatment costs. In this study, crude laccase from the yang1 strain maintained a high decolorization efficiency and enzyme activity even after seven consecutive decolorizations of azo and triphenylmethane dyes. This indicated that crude laccase from the yang1 strain exhibited high stability, strong sustainable degradation capability, and reusability. This characteristic of crude laccase can significantly reduce the cost of efficiently treating actual dye wastewater with laccase. Therefore, crude laccase from the yang1 strain holds significant application value and potential for the efficient treatment of actual dye wastewater. Simultaneously, the stability of crude laccase ensures the reliability and repeatability of the treatment effect, enhancing the overall stability and operational efficiency of the dye wastewater treatment system. This has guiding significance for the large-scale promotion of laccase application in dye wastewater treatment.

### 3.4. Possible Reasons and Mechanisms Behind the Good Detoxification Effect of Crude Laccase from the Yang1 Strain on the Toxicity of Azo and Triphenylmethane Dyes Towards Rice and Wheat Seeds

The results of this study indicated that crude laccase from the yang1 strain exhibited excellent detoxification effects on the toxicity of azo dyes and triphenylmethane dyes on rice and wheat seed germination. The potential causes and mechanisms underlying this phenomenon are analyzed as follows: (1) From the perspective of nutritional metabolism, dye molecules may interfere with the normal nutrient absorption and metabolism processes of plant seeds, thereby affecting seed germination and seedling growth. After treatment with crude laccase, dye molecules may be decomposed into small molecules, and the degradation products are absorbed by plants as energy sources such as carbon, promoting energy metabolism during seed germination and greatly reducing the toxicity of dyes to seed germination. (2) The color of azo dyes and triphenylmethane dyes is primarily determined by the chromophores in their molecules, which are also closely related to the toxicity of the dyes. Laccase can catalyze the oxidation of chromophores in dye molecules, causing structural changes and resulting in dye fading. Laccase may oxidize the -N=N- bond of azo dyes and the triphenylene-conjugated system of triphenylmethane, reducing the dye’s toxicity. In this process, the destruction of chromophores may be accompanied by a decrease in dye toxicity, as toxicity is often related to the specific structure of the molecule.

### 3.5. Compared with Purified Laccase, the Advantages of Using Crude Laccase Solution for Decolorizing Dye Pollutants

Compared with purified laccase, the use of crude laccase solution for treating dye pollutants offers multiple advantages and significant application value. (1) In terms of operational steps, obtaining crude laccase is relatively simple. The purification of laccase involves a series of complex separation and purification steps, such as centrifugation, precipitation, dialysis, and chromatography. These steps not only require professional equipment and technical personnel but also involve cumbersome and time-consuming operations. In contrast, the preparation of crude laccase typically only requires simple fungal culture and preliminary separation processes, greatly simplifying the operational procedure and lowering the technical threshold. This makes crude laccase extremely convenient for the practical treatment of dye wastewater. (2) In terms of application scale, crude laccase is easier to obtain and suitable for large-scale production. Due to the limitations of separation and purification techniques, the yield of purified laccase is often low, making it difficult to meet the demands of large-scale practical applications. However, by optimizing fermentation conditions, it can enhance the laccase production of fungal strains, enabling the large-scale production of crude laccase and thus meeting the needs of large-scale applications such as dye wastewater treatment. (3) In terms of usage cost, crude laccase has a huge cost advantage in practical applications. Due to the elimination of complex purification steps, the production cost of crude laccase is significantly reduced. In the process of treating actual pollutants, the use of crude laccase can greatly reduce the cost of laccase usage, improve economic benefits, and make laccase technology more competitive and valuable in the field of dye wastewater treatment. This is of great significance for promoting the widespread application of laccase in the field of environmental protection and achieving sustainable development.

## 4. Materials and Methods

### 4.1. Fungal Strain and Crude Laccase from Pleurotus Ostreatus Yang1

*Pleurotus ostreatus* yang1 was preserved in Central China Normal University, Wuhan, China [26]. Crude laccase derived from *Pleurotus ostreatus* yang1 was prepared and stored at −20 °C. The preparation procedure was as follows.

An appropriately sized mycelial plug was transferred from the stock plate of the yang1 strain to the center of a Potato Dextrose Agar (PDA) plate and incubated statically at 28 °C. After 7 days of cultivation, when mycelia fully covered the plate, approximately 10 uniformly sized square mycelial blocks were excised from the PDA plate and inoculated into Potato Dextrose Broth (PDB) liquid medium. This was followed by shaking cultivation at 28 °C and 180 rpm for approximately 5 days. After dense and homogeneous mycelial pellets formed in the PDB medium, sterilized 5 mL pipette tips were used to aspirate an appropriate amount of mycelial pellets, which were then transferred to 100 mL of tomato juice medium at an inoculum size of 5% (*v*/*v*). The tomato juice medium was subsequently incubated in a shaking incubator (28 °C, 180 rpm). A previous study has indicated that copper sulfate can enhance laccase expression, serving as an inducer for laccase expression [32]. Therefore, on day 3 of cultivation, laccase-inducing agents (1 mM copper sulfate and 1 mM syringic acid) were added to the tomato juice medium, and shaking cultivation continued. After the addition of the inducer, daily sampling at fixed time intervals was performed to measure laccase activity. When laccase activity reached its peak, the liquid in the tomato juice medium was filtered through gauze to obtain crude laccase. Crude laccase was frozen overnight at −20 °C, thawed at room temperature, and then refiltered through six-layer gauze to yield the final crude enzyme solution. Crude laccase refers to the culture supernatant containing laccase activity, which is preliminarily isolated from fungal cultures with high laccase production. Its main component is laccase.

### 4.2. Dyes Used in This Study

The names, abbreviations, molecular weights, chemical structural formulas, maximum absorption wavelengths, and types of all dyes utilized in this study are presented in Table 1.

### 4.3. Determination of Laccase Activity

Laccase activity was determined using the ABTS (2,2′-Azino-bis-(3-ethylbenzodihydrothiazoline-6-sulfonic acid) diammonium salt) colorimetric method. The reaction system (2 mL) consisted of 200 μL ABTS stock solution (final concentration was 1 mM), 1.6 mL sodium acetate–acetic acid buffer (pH 5.0), and 200 μL enzyme solution to be tested. For the control group, the enzyme solution was replaced with buffer, and the spectrophotometer was zero-adjusted using the control group. The change in absorbance at 420 nm was recorded after 3 min of reaction initiated by adding the enzyme solution in a thermostatic water bath maintained at 30 °C. One unit (U) of laccase activity is defined as the amount of enzyme required to convert 1 µmol of ABTS per minute. The laccase activity (U/L) was calculated using the following formula:

$$U=\frac{\mathrm{Vtotal}\cdot \Delta A\cdot N\cdot 10^{6}}{\mathrm{Venzyme}\cdot \varepsilon \cdot \Delta T}$$

where Vtotal—total volume of the reaction system (L), ΔA—change in absorbance, N—sample dilution factor, Venzyme—volume of enzyme solution (L), ε—molar extinction coefficient of ABTS (ε = 3.6 × 10^4^ M^−1^ cm^−1^), and ΔT—reaction time (min).

### 4.4. Decolorization of Azo and Triphenylmethane Dyes by Crude Laccase and Calculation of Decolorization Efficiency

The 1 mL decolorization reaction system contained yang1 crude laccase (1 U/mL or 2 U/mL) and dye at final concentrations of 50, 100, 200, 400, 800, or 1000 mg/L, supplemented with sodium acetate–acetic acid buffer (pH 5.0) to a total volume of 1 mL. The control group contained no laccase. After thorough mixing, the reaction proceeded for 24 h at 30 °C in a light-proof thermostatic water bath. The absorbance at the maximum absorption wavelength of the dye was then measured using an ultra-micro spectrophotometer. Decolorization efficiency (%) was calculated as follows:

$$\mathrm{Decolorization}\;\mathrm{efficiency}\left(\%\right)=\frac{\left(A0-\mathrm{At}\right)}{A0}\times 100\%$$

where A0 represents the initial absorbance and At represents the absorbance after 24 h of decolorization.

### 4.5. Time Course Decolorization of Different Dyes by Crude Laccase

The 1 mL decolorization reaction system contained yang1 crude laccase (1 U/mL) and dye at final concentrations of 25, 50, 100, 200, or 400 mg/L, supplemented with sodium acetate–acetic acid buffer (pH 5.0) to a total volume of 1 mL. After thorough mixing, the reaction proceeded at 30 °C in a light-proof thermostatic water bath. Samples were collected at reaction times t = 0, 0.5, 1, 1.5, 2, 3, 6, 9, 12, and 24 h. Absorbance was measured at each time point, and decolorization efficiency was calculated to generate time course decolorization curves. The decolorization efficiency calculation formula is identical to that described in Section 2.4.

### 4.6. Kinetic Study of Dye Degradation by Crude Laccase

Dye solutions with a concentration of 100 mg/L were prepared. Absorbance (A) was measured at the maximum absorption wavelength to establish an A-c standard curve. The residual dye concentration ct in the system at different reaction times was calculated. When the reaction rate is directly proportional only to the first power of the dye concentration, the reaction is classified as first-order. The relationship between the reaction rate and dye concentration can be expressed as ln ct=−kt+lnc0. A plot of ln c versus time (t) yields the ln c–t curve. The reaction rate constant $k=\ln\frac{c0}{\mathrm{ct}}$, and the negative slope of the straight line on the ln c–t plot corresponds to the reaction rate constant k. Here, c0 represents the initial concentration, ct represents the concentration at time t, and k is the reaction rate constant.

### 4.7. Decolorization of Azo + Triphenylmethane Dye Mixtures by Crude Laccase—Determination of Individual Dye Decolorization Efficiency

The 1 mL decolorization reaction system contained yang1 crude laccase (1 U/mL or 2 U/mL) and both azo and triphenylmethane dyes at final concentrations of 50, 100, 200, 400, 800, or 1000 mg/L each, supplemented with sodium acetate–acetic acid buffer (pH 5.0) to a total volume of 1 mL. The control group contained no laccase. After thorough mixing, the reaction proceeded for 24 h at 30 °C in a light-proof thermostatic water bath. The absorbance at the maximum absorption wavelength of each individual dye within the mixture was measured using an ultra-micro spectrophotometer, and the decolorization efficiency for each dye was calculated. The decolorization efficiency calculation formula is identical to that described in Section 2.4.

### 4.8. Sequential Batch Decolorization of Azo and Triphenylmethane Dyes by Crude Laccase

The sequential decolorization reaction system volume was 1 mL in volume, containing yang1 crude laccase (1 U/mL), dye solution (100 mg/L), and 50 mM acetate buffer (pH 5.0). The reaction was conducted at 30 °C. Each decolorization cycle lasted 24 h, and the process was repeated consecutively for 7 cycles. The absorbance of the dye in a reaction system without added laccase (A10) served as the cycle 1 control. After 24 h incubation, the absorbance of the experimental group at the maximum absorption wavelength (A11) was measured using an ultra-micro spectrophotometer. The decolorization efficiency D1 for cycle 1 was calculated from the change in absorbance. Immediately after the 24 h reaction, a fresh 100 mg/L dye solution (without additional laccase) was added to the same reaction mixture. After mixing, the absorbance was measured immediately to serve as the cycle 2 control (A20). The reaction continued at 30 °C for another 24 h, after which the absorbance (A21) was measured, and the decolorization efficiency for cycle 2 (D2) was calculated. This process continued for 7 consecutive cycles. The decolorization efficiency of cycle 1 was set as 100%, and the relative decolorization percentage for each subsequent cycle was calculated. The formulas for decolorization efficiency (Dt%) and relative decolorization percentage (Rt%) are as follows:

$$\mathrm{Dt}\%=\frac{\mathrm{At}0-\mathrm{At}1}{\mathrm{At}0}\times 100\%$$

$$\mathrm{Rt}\%=\frac{\mathrm{Dt}}{D1}\times 100\%$$

where Dt—decolorization efficiency of cycle t, At0—absorbance before decolorization in cycle t, At1—absorbance after decolorization in cycle t, D1—decolorization efficiency of cycle 1, Dt—decolorization efficiency of cycle t, and t—cycle number.

### 4.9. Detoxification of Plant Toxicity by Crude Laccase on Azo Dyes, Triphenylmethane Dyes, and Mixtures of Azo and Triphenylmethane Dyes

Rice and wheat seeds were pretreated by washing 2–3 times with tap water, and plump seeds were selected for soaking (rice seeds for 24 h, wheat seeds for 12 h). Subsequently, 20 plump, healthy seeds of uniform size were placed in Petri dishes lined with filter paper. A blank control group, control group, and experimental group were established, each with a 5 mL system. The blank control group contained only 5-fold diluted 50 mM sodium acetate–acetic acid buffer (without any dye). The control group contained either single dyes at final concentrations of 50, 100, 200, 400, 800, or 1000 mg/L, or dye mixtures at final concentrations of 50 + 50, 100 + 100, 200 + 200, 400 + 400, 800 + 800, or 1000 + 1000 mg/L, mixed with 5-fold diluted 50 mM sodium acetate–acetic acid buffer (without laccase). The experimental group contained either single dyes (50–1000 mg/L) or dye mixtures (50 + 50 − 1000 + 1000 mg/L), mixed with 5-fold diluted 50 mM sodium acetate–acetic acid buffer and 1 U/mL crude laccase. All three groups were incubated at 30 °C in darkness for 24 h. After incubation, the 5 mL solutions from the blank control, control, and experimental groups were poured into their respective Petri dishes to soak the seeds. The dishes were placed in a 30 °C incubator for cultivation (wheat seeds for 3 days, rice seeds for 4 days), after which shoot length and root length were measured.

### 4.10. Molecular Docking of Yang1 Strain Laccase LAC-Yang1 and Dyes

#### 4.10.1. Homology Modeling of Yang1 Strain Laccase LAC-Yang1 with Known Amino Acid Sequence

Our laboratory’s preliminary research obtained the amino acid sequence of LAC-Yang1 laccase from *Pleurotus ostreatus* yang1. We searched and viewed the complete amino acid sequence of laccase in the UniProt database (www.uniprot.org) and compared it using the inbuilt sequence comparison tool. We then used SWISS-model (https://swissmodel.expasy.org/interactive (accessed on 2 August 2025)) in automated mode for the construction of the laccase 3D structure and to remove the original ligands and water molecules of the laccase by using PyMOL. The laccase 3D structure is shown in Figure S1.

#### 4.10.2. Optimization of Dye Structure

The structures of the dye molecules were obtained from the PubChem database (https://pubchem.ncbi.nlm.nih.gov/ (accessed on 11 February 2025)). We imported the downloaded dye structures into PyMOL to remove the original ligands such as metal ions present in the dye molecules and processed them for minimum energy optimization using Chem3D 21.0.0.

#### 4.10.3. Molecular Docking of Yang1 Strain Laccase LAC-Yang1 with Dyes

Two types of molecular docking software (Molegro Virtual Docker 2013.6.0.1-MVD and Autodock 4.2.6) are utilized for the molecular docking between laccase and dyes.

Molegro Virtual Docker 2013.6.0.1 (MVD): we used Molegro Virtual Docker (MVD) with optimized dye structures as ligands and yang1 strain laccase LAC-Yang1 as docking acceptors, and applied a high-precision lattice algorithm to predict the binding sites of proteins and ligands. The ligand was placed in all possible docking pockets of the protein in order to search for the best docking site. To improve the docking accuracy, we chose the search algorithm as MolDock SE for 50 iterative operations, and the other parameters involved were set to default values. Finally, we verified the docking accuracy using the re-docking method to determine the best conformations for laccase–dye binding according to the MolDock score.

Autodock 4.2.6: Autodock 4.2.6 was used to perform molecular docking experiments with laccase and the dyes. Laccase and ligands were processed and saved in pdbqt format according to the needs of the experiment. The size of the docked lattice was 100 Å × 100 Å × 100Å. Docking was performed using semi-flexible docking. Autogrid was used to calculate the lattice point energy. Docking operations were run 50 times using the Lamarckian Genetic Algorithm (LGA). Laccase–dye binding was evaluated using a semi-empirical free energy calculation with other parameters set to default values. The appropriate conformation was selected based on the minimum binding energy.

#### 4.10.4. Analysis of Molecular Docking Results

The molecular docking results were visualized and analyzed using PyMOL 3.1.3.1 and LigPlot+ v.2.2.9. PyMOL can generate 3D images of protein and ligand bound conformations, and LigPlot+, a graphical program that generates graphs of the interaction between proteins and ligands, can be used to study the binding modes of both. The hydrogen bonding and hydrophobic interactions of laccase and the dyes were evaluated using PyMOL and LigPlot+, and 3D and 2D interaction diagrams showing information on the amino acid residues of the proteins involved in the hydrogen bonding and hydrophobic interactions as well as the lengths of hydrogen bond were generated, which provided an in-depth insight into the details of the binding modes of laccases and dyes of different structures.

### 4.11. Statistical Analysis of Experimental Data

All experiments were performed in triplicate with 3 sample replicates. Data were presented as mean ± standard deviation. Microsoft Excel was used to calculate the data according to the corresponding formula. Graphpad prism 10 was used to calculate the mean value and standard deviation of the data and plots and was also used to test the difference between the two groups of means to determine whether there was a significant difference between the two groups. ns meant no significant difference between groups, *p* > 0.05; * meant a significant difference between groups, *p* ≤ 0.05; ** meant a highly significant difference between groups, *p* ≤ 0.01; *** meant an extremely significant difference between groups, *p* ≤ 0.001; and **** meant an exceedingly significant difference between groups, *p* ≤ 0.0001.

## 5. Conclusions

Crude laccase from the yang1 strain showed a high decolorizing capacity against various azo dyes and triphenylmethane dyes. The decolorization efficiency improved significantly as the laccase dosage increased. The decolorization process of laccase on triphenylmethane dyes conformed to the first-order reaction kinetics model, with the ranking of reaction rate constants k as follows: BB > MG > CR > AG50 > BB1 > EV > BB7. Crude laccase demonstrated a significant continuous decolorization ability for the azo dyes α-NO and MB13, according to the results of the continuous batch decolorization experiments. Additionally, crude laccase also demonstrated a good decolorization performance on continuous batches of triphenylmethane dyes, such as CR and AG50, with laccase activity remaining relatively stable during the decolorization process. Although the activity of crude laccase decreased after multiple cycles, its decolorizing ability for specific dyes remained strong. Crude laccase exhibited good reusability and sustainable degradability in the continuous decolorization process of dyes. The decolorization by laccase demonstrated remarkable detoxification capabilities against the toxicity posed by both single azo dyes and single triphenylmethane dyes to rice and wheat seeds. Following treatment with crude laccase, the toxicity of azo dyes DB2 and TO, as well as triphenylmethane dyes BB1, BB7, EV, and CR, towards rice and wheat seeds was significantly reduced or even completely eliminated. Crude laccase also showed good decolorization effect on a mixture of azo dyes and triphenylmethane dyes (mixture of two types of dyes) and had a strong detoxification ability for the phytotoxicity of mixed dyes. The results of molecular docking studies between laccase and different structural dyes revealed the interaction mechanism between laccase protein and dye molecules, as well as the reasons and possible mechanisms for laccase’s differences in catalytic ability on different structural dyes from a theoretical perspective. In summary, crude laccase from the yang1 strain demonstrated remarkable decolorizing effects and excellent detoxifying capabilities on various single dyes and mixed dyes. It possessed great application value and potential for efficiently degrading and detoxifying dye pollutants of different structural types.

## Figures and Tables

**Table 1:** Names, abbreviations, molecular weights, chemical structures, maximum absorption wavelengths, and types of all dyes used in this study.

Name	Abbreviation	Molecule Weight (g/mol)	Structure	Maximum Absorbance Wavelength (nm)	Type
Acid Orange 7	AO7	350.3	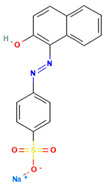	481	Azo dye
Acid Red 1	AR1	509.4	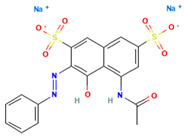	505	Azo dye
Direct Blue 2	DB2	830.7	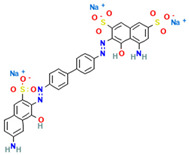	566	Azo dye
Mordant Blue 13	MB13	518.8	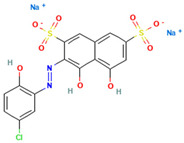	522	Azo dye
Orange G	OG	452.4	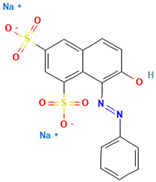	478	Azo dye
Tropaeolin O	TO	316.2	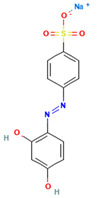	387	Azo dye
Alpha-Naphthol Orange	α-NO	350.3	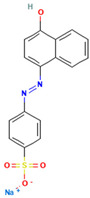	473	Azo dye
Acid Green 50	AG50	576.6	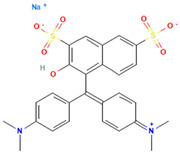	636	Triphenylmethane dye
Bromophenol Blue	BB	670.0	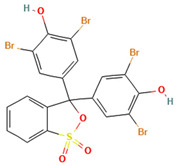	591	Triphenylmethane dye
Basic Blue 1	BB1	399.4	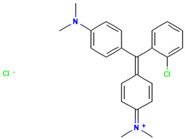	631	Triphenylmethane dye
Basic Blue 7	BB7	514.1	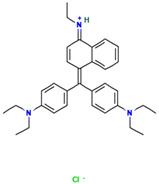	555	Triphenylmethane dye
Brilliant Blue G	BBG	854.0	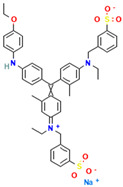	581	Triphenylmethane dye
Cresol Red	CR	382.4	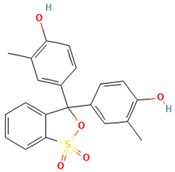	435	Triphenylmethane dye
Ethyl Violet	EV	492.1	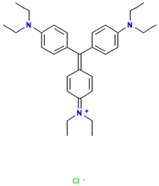	542	Triphenylmethane dye
Methyl Green	MG	653.2	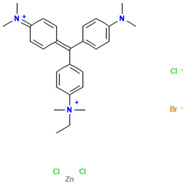	632	Triphenylmethane dye

**Table 2:** Comparison between results of decolorization efficiencies of azo dyes by the laccase and molecular docking results (HBond: Hydrogen bonding energy).

Name	Dye Concentration (mg/L)			MolDock Score	Re-Rank Score	HBond (kJ/mol)	Binding Affinity (kJ/mol)
	10	25	50				
AO7	82%	92%	88%	−114.519	−95.4339	−5.82743	−24.0421
AR1	89%	75%	66%	−114.354	−96.3192	−9.50236	−22.0926
DB2	100%	83%	71%	−152.437	−111.212	−6.91448	−38.4061
MB13	93%	94%	92%	−118.308	−90.0838	−12.877	−23.5246
α-NO	100%	92%	95%	−121.386	−48.5297	−6.63471	−24.925
TO+	51%	47%	34%	−107.139	−70.8275	−0.46999	−33.1705
TO-				−69.5667	−61.6063	−3.45049	−21.8967

**Table 3:** Statistics on the number of interactions between azo dyes and laccase.

Ligand	Hydrophobic Interaction	Representative Hydrophobic Interaction	Hydrogen Bond
AO7	54	10	5
AR1	53	10	8
DB2	86	16	4
MB13	55	8	7
α-NO	77	11	6
TO+	39	10	0
TO-	21	7	2

**Table 4:** Comparison between results of decolorization efficiencies of triphenylmethane dyes by the laccase and molecular docking results. HBond: Hydrogen bonding energy. “—” represents not decolorization.

Name	Dye Concentration (mg/L)			MolDock Score	Re-Rank Score	HBond (kJ/mol)	Binding Affinity (kJ/mol)
	10	25	50				
AR92	—	—	—	−113.844	−68.075	−4.02983	−56.0656
AG50	80%	64%	62%	−130.848	−89.8571	−8.28465	−15.8195
CR	51%	54%	57%	−128.55	−21.4867	0.504288	−22.5065
BB	97%	100%	100%	−116.665	−72.0983	−3.62004	−38.4771
MG	99%	98%	98%	−108.117	−78.5417	−0.405233	−18.2366
BB1	88%	78%	73%	−113.282	−88.3131	−0.378087	−22.6697
EV	75%	76%	73%	−129.258	−72.5065	0	−25.3519
BB7	33%	71%	76%	−125.383	−86.5739	−0.436326	−31.3687

**Table 5:** Statistics on the number of interactions between triphenylmethane dyes and laccase.

Ligand	Hydrophobic Interaction	Representative Hydrophobic Interaction	Hydrogen Bond
AR92	35	4	5
AG50	54	13	4
CR	88	10	3
BB	42	6	4
MG	47	13	0
BB1	44	14	0
EV	56	17	0
BB7	57	14	0

## Data Availability

The data presented in this study are available in article.

## Supplementary Materials

The following supporting information can be downloaded at: https://www.mdpi.com/article/10.3390/ijms26178363/s1.
