# Supplementary figures and images for: Decolorization and Detoxification of Azo and Triphenylmethane Dyes Damaging Human Health by Crude Laccase from White-Rot Fungus Pleurotus ostreatus Yang1 and Molecular Docking Between Laccase and Structurally Diverse Dyes
Source: Int J Mol Sci. 2025 Aug 28;26(17):8363. doi: 10.3390/ijms26178363 (PMC12428645; doi:10.3390/ijms26178363)

**Figure S1. 3D structure of LAC-Yang1 laccase.**

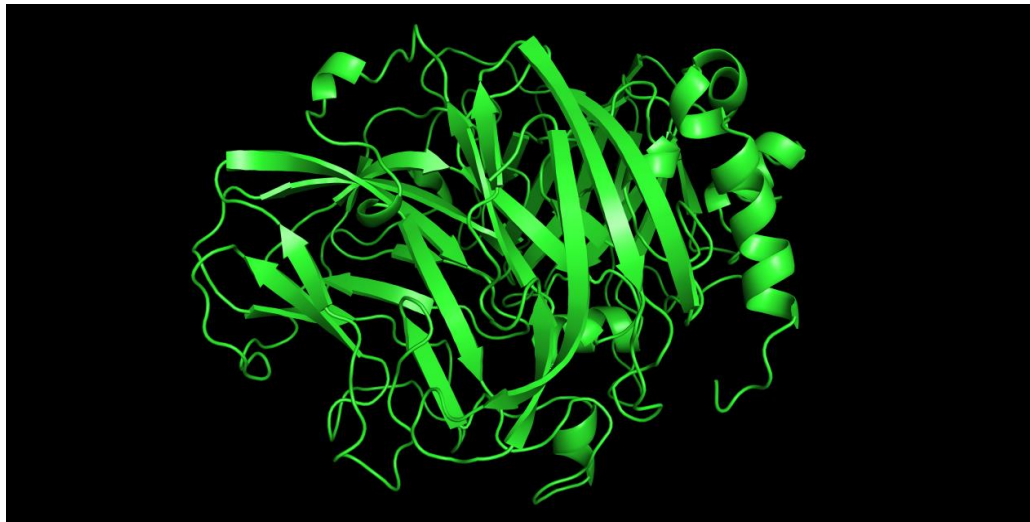

Supplement: Supplementary file 1 [file ijms-26-08363-s001.zip › ijms-3798870-supplementary.pdf]
